# Sleep duration and compliance with sleep recommendations among Brazilian children and adolescents: an updated systematic review and meta-analysis

**DOI:** 10.1016/j.jped.2026.101608

**Published:** 2026-09-16

**Authors:** Gabriel Pereira Maciel, Ricardo de Camargo, Maria Beatriz Vieira de Brito, Gabrielli Thais de Mello, Valter Cordeiro Barbosa Filho, Kelly Samara Silva

**Affiliations:** a Universidade Federal de Santa Catarina (UFSC), Post-graduate Program in Physical Education, Florianópolis, SC, Brazil; b Universidade Federal de Santa Catarina (UFSC), Multicenter Graduate Program in Physiological Sciences, Florianópolis, SC, Brazil; c Institute for Advancing Health through Agriculture (IHA), Texas A&M AgriLife Research, Dallas, TX, United States; d Universidade Estadual do Ceará (UECE), Post-Graduate Program on Collective Health, Fortaleza, CE, Brazil; e Universidade Federal de Santa Catarina (UFSC), Department of Physical Education, Florianópolis, SC, Brazil

**Keywords:** Sleep duration, Youth, Childhood, Prevalence, Meta-analysis, Brazil

## Abstract

**Objective:**

To update the evidence on sleep duration among children and adolescents, provide pooled estimates of mean sleep duration and compliance with sleep recommendations, and examine whether differences in measurement methods, assessment instruments, and guideline criteria contribute to variability in reported estimates.

**Sources:**

Eight databases (PubMed, PsycINFO, CINAHL, SciELO, Embase, Web of Science, Scopus, and LILACS) were searched from January 2020 to June 2025, supplemented by studies identified in a previous review. Population- or school-based observational studies of participants aged 0–18 years that reported sleep duration were eligible. Random-effects meta-analyses were conducted, and subgroup analyses and meta-regression were used to explore heterogeneity.

**Summary of the findings:**

Eighty-four studies involving 177,074 participants were included. The pooled prevalence of adequate sleep duration was 45% (95% CI, 29–62%) among preschoolers, 65% (95% CI, 55–74%) among school-age children, and 47% (95% CI, 40–55%) among adolescents. Mean sleep duration decreased with age, ranging from 10.50 h (95%CI, 9.85–11.20) in preschoolers to 7.77 h (95%CI, 7.42–8.12) in adolescents. Questionnaire-based measures yielded higher estimates than accelerometry, and prevalence varied by the sleep guideline adopted. Heterogeneity was substantial across analyses (I² > 98%).

**Conclusions:**

Many Brazilian children and adolescents do not achieve recommended sleep duration, which can compromise their health and development. Although the pooled estimates should be interpreted with caution due to substantial between-study heterogeneity, the findings highlight the need for standardized sleep assessment and reporting to improve comparability across studies and strengthen epidemiological surveillance and public health planning.

## Introduction

Sleep is an essential component of children’s and adolescents’ health, with implications for psychological, cognitive, and physical outcomes [1,2]. Sleep health is a multidimensional construct encompassing several interrelated domains, including sleep duration, timing and regularity, efficiency, satisfaction or quality, and daytime alertness [2]. Among these dimensions, sleep duration is the most commonly assessed dimension of sleep health and generally declines from early childhood through adolescence [3]. Adolescents are particularly vulnerable to insufficient sleep because circadian phase delays promote later bedtimes, while early school schedules and social demands may limit sleep opportunities [4,5].

Sleep duration recommendations are therefore age-specific. Guidelines from the American Academy of Sleep Medicine (AASM) [6], the National Sleep Foundation (NSF) [7], and the Canadian 24-Hour Movement Guidelines [8] consistently recommend progressively shorter sleep durations from childhood to adolescence, although their age ranges and cut-off points differ slightly. Epidemiological studies indicate that insufficient sleep is common and becomes increasingly prevalent during adolescence. In the United States, survey-based estimates derived from parent reports or child self-reports indicated that 34.9% of children slept less than recommended [9], while Canadian self-reported survey data indicated that approximately 26%–31% of youth aged 10–17 years were short sleepers [10]. Device-based data from Spain further showed that adherence to recommendations dropped from 33.7% at age 12 to 18.7% at age 16 [11].

Although these international findings demonstrate that insufficient sleep is common among children and adolescents, Brazilian evidence suggests that sleep duration may vary according to socioeconomic characteristics, regional and school schedules, particularly morning school attendance [12]. However, the available Brazilian studies remain fragmented and geographically uneven, restricting comparisons across regions and over time. In addition to these contextual and population-level differences, methodological variation further complicates the interpretation and comparison of sleep-duration estimates across studies. Sleep duration may be assessed using questionnaires, diaries, accelerometers, or other devices, with differences in the indicators derived, reference periods considered, and definitions applied [13,14]. Subjective methods are particularly common and may produce estimates that differ from those obtained using device-based measures [14]. Studies also differ in whether they assess time in bed or actual sleep duration, whether they distinguish between school and non-school days, and whether they apply age-specific cut-off points from different guidelines or study-defined criteria [1].

This issue is especially relevant in Brazil, where the available epidemiological evidence is scattered across studies that use different measurement instruments and criteria for defining adequate sleep duration. Moreover, Brazil lacks national sleep-duration guidelines, which may contribute to inconsistencies in the classification and reporting of sleep adequacy. The previous systematic review did not systematically examine whether measurement methods and guideline criteria were associated with differences in reported estimates [15]. Therefore, an updated quantitative synthesis is needed to estimate the magnitude of insufficient sleep, assess the consistency of findings, and support the interpretation of epidemiological evidence. From this perspective, this study aimed to update the evidence on sleep duration among children and adolescents, provide pooled estimates of mean sleep duration and compliance with sleep recommendations, and examine whether differences in measurement methods, assessment instruments, and guideline criteria contribute to variability in reported estimates. By quantitatively synthesizing the available evidence and exploring potential sources of heterogeneity, this review seeks to improve the interpretation of sleep-duration estimates in Brazil.

## Methods

This systematic review was written in accordance with the Preferred Reporting Items for Systematic Reviews and Meta-Analyses (PRISMA) statement [16]. As this review updates a previous systematic review [15], only studies published from 2020 onwards were included in the combined review. This systematic review was developed as part of the Brazilian Report Card initiative, which participates in the Global Matrix on Physical Activity for Children and Adolescents. Although sleep is not currently a core Global Matrix indicator, it was the most frequently suggested additional indicator for consideration in future editions [17].

### Eligibility criteria

Studies were considered eligible if they met the following criteria: (1) original research articles published in peer-reviewed journals; (2) studies including samples of Brazilian children and adolescents aged 0–18 years (or with a mean age within this range, or reporting results separately for this age group); (3) studies reporting sleep duration variables (e.g., hours per night, minutes per night, or prevalence of adequate sleep duration, regardless of the cut-off adopted), measured using either objective methods (e.g., polysomnography or actigraphy) or subjective methods (e.g., self-report, proxy report, or parent/guardian report); (4) population-based or school-based surveys employing probabilistic or random sampling procedures, ensuring representativeness of the target population; and (5) observational study designs, including cross-sectional or cohort studies.

No restrictions were applied regarding language. Studies were excluded if they: (1) included exclusively participants with diagnosed sleep disorders (e.g., insomnia or sleep apnea); (2) were based on convenience samples; or (3) focused exclusively on participants recruited on the basis of a specific disease or health condition that could affect the health estimates, such as obesity or diabetes. Clinically selected samples are associated with a higher prevalence of short sleep, and clinical recruitment could therefore bias population estimates toward poorer sleep and limit their generalizability [18]. Population-based or school-based studies were not excluded when participants with these conditions were naturally represented within the sampled population.

### Information sources

The literature search was conducted on June 13, 2025, across the following electronic bibliographic databases: PubMed (NCBI), PsycINFO, CINAHL (EBSCOhost), SciELO, Embase (Elsevier), Web of Science (Clarivate), Scopus (Elsevier), and LILACS. All searches were performed through the CAPES Journal Portal (*Portal de Periódicos da CAPES*), which provides institutional access to multiple scientific databases. Databases were searched from January 2020 to June 13, 2025.

### Search strategy

The search strategy was structured around four groups of descriptor terms: (1) sleep-related, (2) context-related, (3) population-related, and (4) terms on outcome assessment. Boolean operators were used to combine search terms, with “AND” applied between descriptor groups and “OR” applied within each group. Database-specific truncation symbols (e.g., *, $, or quotation marks) were used to expand the search to include variations of the descriptors.

Search strategies were developed in English and Portuguese for databases that index Portuguese descriptors (i.e., LILACS and SciELO). The complete search strategies for each database are provided in Supplementary Material 1.

### Selection process

The study selection process was conducted in two stages using Rayyan, a web-based systematic review tool. First, the titles and abstracts of all retrieved records were screened to identify potentially eligible studies. When eligibility could not be determined solely from the title and abstract, the full text was consulted. In the second stage, the full texts of potentially relevant articles were assessed against the predefined eligibility criteria.

**Figure 1:**
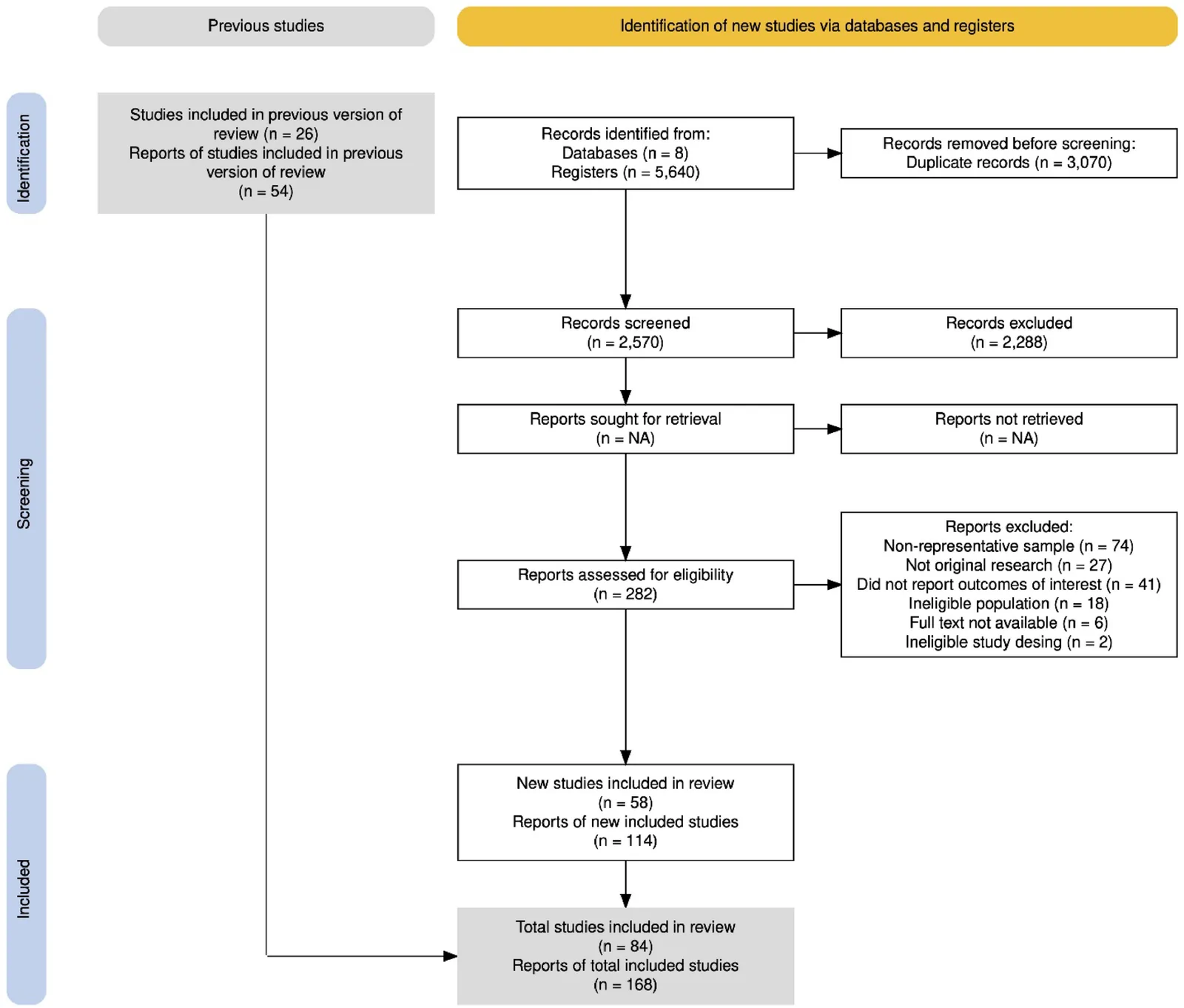
PRISMA flow diagram for updated systematic review of the study selection process.

All screening procedures were performed independently by two pairs of reviewers (pair 1: GPM and RC; pair 2: MB and GTM). Each pair screened approximately 50% of the retrieved records. Disagreements between reviewers were resolved through discussion and consensus among all reviewers. The selection process is presented in the PRISMA flow diagram (Figure 1) [19].

### Data collection process

Data extraction was performed independently by the four reviewers involved in the screening process (GPM, RC, MB, and GTM) using a standardized data extraction form developed for this review. Each study had data extracted and subsequently checked by another reviewer to ensure accuracy and consistency. Any disagreements between reviewers were resolved through discussion and consensus.

The extraction form included predefined variables related to study and sample characteristics, as well as sleep duration outcomes. When relevant information required for the analyses was missing (e.g., standard deviations or sample size for prevalence estimates), the corresponding authors of the studies were contacted to obtain or confirm the data.

### Data item (outcomes)

The primary outcomes of interest were sleep duration and compliance with recommendations for sleep duration, such as those from the American Academy of Sleep Medicine (AASM) [6], the National Sleep Foundation [7], and the Canadian Guideline [8].

For the continuous outcome, data were extracted on the mean sleep duration (hours per day) and its corresponding measures of variability (standard deviation or confidence intervals). When studies reported median and interquartile range instead of mean and standard deviation, these statistics were also extracted for potential conversion.

For the categorical outcome, data were extracted on the prevalence of adequate sleep duration, as defined in each study. Information on the guideline used (e.g., AASM [6], National Sleep Foundation [7], Canadian Guideline[8]) and the minimum and maximum recommended sleep duration thresholds were recorded when available.

When multiple estimates were reported within a study (e.g., by sex, age group, or other subgroups), all relevant results compatible with the outcome domains were extracted.

When necessary, additional calculations were performed to obtain the required summary statistics. For example, when studies reported sleep duration using confidence intervals or medians with interquartile ranges, these statistics were converted into means and standard deviations when possible. For categorical outcomes, prevalence estimates were extracted directly when reported. When studies reported only the prevalence or number of participants who did not meet sleep-duration recommendations, the corresponding proportion meeting the recommendations was calculated as the complement of that category, using the same denominator. No assumptions regarding the distribution of participants across additional sleep-duration categories were required.

### Data items (other variables)

Additional data extracted from each study included publication characteristics (authors, year of publication, study title), study characteristics (study design, study name, location and region in Brazil, and year of data collection), and sample characteristics (sample size, percentage of girls, mean age, standard deviation of age, and reported age range). Age was categorized into preschoolers (< 5 lt; 5 years), school-age (5–13 years), and adolescents (14–18 years).

Information regarding sleep assessment methods was also extracted, including the type of instrument used (e.g., accelerometer, questionnaire, or other), the name of the instrument when reported, and whether the instrument was clearly defined in the study classified as “defined” when a valid and established questionnaire was nominated (e.g., Pittsburgh Sleep Quality Index), “undefined” when no information regarding the sleep duration measurement was provided, or “undefined-reproductible” when the questionnaire was not nominated but authors fully described the questions used for assessing sleep duration.

### Risk of bias assessment

Risk of bias was assessed using the Newcastle–Ottawa Scale adapted for descriptive cross-sectional studies (NOS-xs2) [20]. Although other tools are available for prevalence studies, the NOS-xs2 was selected because it was specifically developed for descriptive cross-sectional studies and closely matched the characteristics of the evidence included in this review. Many included studies reported only mean sleep duration or the prevalence of meeting sleep-duration recommendations, without examining exposure–outcome associations or presenting adjusted regression models. The NOS-xs2 comprises two domains and three items directly relevant to this type of evidence: study-sample selection, assessed through representativeness and sample-size adequacy, and outcome assessment.

Because this review was conducted within the Brazilian Report Card initiative and aimed to provide population-level estimates relevant to surveillance, the eligibility criteria were deliberately restricted to population- or school-based studies that used probabilistic or random sampling procedures and included samples considered adequate for estimating population prevalence. These criteria were aligned with international recommendations for movement-behaviour surveillance, which emphasize the use of representative samples of sufficient size to characterize population estimates and inequalities [21]. Consequently, all included studies met the predefined criteria for representativeness and sample-size adequacy and received one star for each of these two items.

For outcome assessment, polysomnography was predefined as the gold-standard method and was awarded two stars; however, none of the included studies used it. One star was awarded when sleep duration was assessed using accelerometry, a validated questionnaire, or a sufficiently detailed description of the question used to measure the outcome, allowing the assessment to be reproduced. Studies that did not identify or describe the sleep-duration assessment method received zero stars.

The NOS-xs2 score ranged from zero to four stars. Studies receiving three or four stars were classified as having a low risk of bias, those receiving two stars as having a moderate risk of bias, and those receiving one or zero stars as having a high risk of bias. Because representativeness and sample-size adequacy were incorporated into the eligibility criteria, the minimum possible score among the included studies was two stars. Thus, within this deliberately restricted evidence base, the assessment distinguished between moderate and low risk of bias primarily according to the quality and reporting of the sleep-duration measurement.

### Effect measures and synthesis methods

For the synthesis, studies were grouped according to the type of outcome reported, including (i) studies reporting mean sleep duration (continuous outcome) and (ii) studies reporting the prevalence of adequate sleep duration (categorical outcome). For the continuous outcome, sleep duration was summarized as the pooled mean (hours per day) with corresponding 95% confidence intervals (95% CI). For the categorical outcome, the prevalence of adequate sleep duration was used as the effect measure and expressed as proportions with corresponding 95% confidence intervals (95% CI), based on the definitions of adequate sleep duration adopted in each study. Only studies providing sufficient statistical information to calculate the respective effect measures were included in each synthesis.

Study characteristics and extracted outcomes were summarized in descriptive tables. Forest plots were used to visually display the results of individual studies and the pooled estimates from the meta-analyses.

Meta-analyses with random-effects models were used to estimate the pooled mean sleep duration and the pooled prevalence of adequate sleep duration, accounting for expected methodological and population heterogeneity across studies. For prevalence outcomes, proportions were pooled using a logit transformation to stabilize variances. Continuous outcomes were synthesized using inverse-variance weighting.

Age-group categories were pre-specified as part of the Brazilian Report Card methodology and were incorporated into the data-extraction process. As a preliminary step, overall random-effects meta-analyses including all eligible studies were conducted for prevalence and mean sleep duration. The pooled prevalence was 54% (95% CI: 47%–60%; I² = 99.7%; τ² = 1.13), and the mean sleep duration was 9.01 h per night (95% CI: 8.71–9.46; I² = 99.9%; τ² = 2.37). Age group was then evaluated as a moderator using meta-regression and was statistically significant for the prevalence estimate (QM = 26.29, p = 0.0002; 23.70% of the between-study heterogeneity) and the mean estimate (QM = 60.82, p < 0.0001; 48.52% of the between-study heterogeneity). Also, the health impacts and determinants of sleep differ across life stages. Thus, all analyses were performed for each age group to address clinical heterogeneity.

Statistical heterogeneity among studies was assessed using the Cochran Q test and quantified using the I² statistic. Between-study variance (τ²) was estimated using the restricted maximum likelihood (REML) method. Given the substantial between-study heterogeneity, 95% prediction intervals were calculated for each age-specific random-effects model. Because age group was identified as a significant moderator in the preliminary analyses, prediction intervals were estimated separately for preschoolers, school-age children, and adolescents, consistent with the primary analytical approach. These intervals represent the expected range of estimates in a future comparable study within the same age group. For prevalence outcomes modelled on the logit scale, prediction limits were back-transformed and presented as percentages.

To explore potential sources of heterogeneity, subgroup analyses were conducted according to predefined study characteristics, including sex, type of sleep assessment instrument, sleep recommendation guideline used to define adequate sleep duration, and risk of bias.

Given the substantial heterogeneity observed across studies, meta-regression analyses were conducted to explore potential sources of variability in the pooled estimates. Study-level variables examined in the meta-regression included risk of bias and the guideline used to define adequate sleep duration for the prevalence outcome, and risk of bias and the measurement instrument for the continuous outcome.

Potential reporting bias was assessed through visual inspection of funnel plots and statistical evaluation using Egger’s regression test. Funnel plots were used to examine asymmetry in the distribution of study estimates, which may indicate small-study effects or publication bias. Egger’s test was applied to assess funnel plot asymmetry when sufficient studies were available. All analyses were conducted using the meta package in R.

## Results

### Study selection

A total of 5640 records were identified through database searches. After removing 3070 duplicate records, 2570 records remained for title and abstract screening. Of these, 2288 records were excluded because they did not address the outcome of this review. Of the 282 reports in the full-text evaluation, 168 were excluded. Thus, 58 studies[[22], [23], [24], [25], [26], [27], [28], [29], [30], [31], [32], [33], [34], [35], [36], [37], [38], [39], [40], [41], [42], [43], [44], [45], [46], [47], [48], [49], [50], [51], [52], [53], [54], [55], [56], [57], [58], [59], [60], [61], [62], [63], [64], [65], [66], [67], [68], [69], [70], [71], [72], [73], [74], [75], [76], [77], [78], [79]] (114 reports) were included in the update. Considering the 26 studies[[80], [81], [82], [83], [84], [85], [86], [87], [88], [89], [90], [91], [92], [93], [94], [95], [96], [97], [98], [99], [100], [101], [102], [103]] (54 reports) from the previous systematic review, 84 studies (168 reports) were summarized.

### Study characteristics

A total of 84 studies were included in this review. Most studies focused on school-age children and adolescents (n = 36 each), with a smaller number including preschoolers (n = 12) (Table 1, and detailed information for all studies is provided in Supplementary Table S1). Most studies were cross-sectional (n = 75) and were conducted in the South (n = 37), Northeast (n = 24), or Southeast (n = 21), whereas only two provided nationwide estimates. Questionnaires were the predominant measurement method (n = 78), with only six studies using accelerometers. The main characteristics of the included studies are summarized in Table 1, with study-level information provided in Supplementary Table S1.

**Table 1:** Characteristics of the included studies (84 studies).

Category	Total(n)	Preschoolers (< 5 years) (n)	School-age Children (5–13 years) (n)	Adolescents (14–18 years) (n)
Included studies	84	12	36	36
Study design				
Cross-sectional	75	9	32	34
Longitudinal	9	3	4	2
Region				
South	37	2	20	15
Northeast	24	8	4	12
Southeast	21	1	12	8
Country-wide	2	1	0	1
Measurement method				
Questionnaire	78	12	34	32
Accelerometer	6	0	2	4
Outcome reporting				
Categorical only	41	1	19	21
Continuous only	31	5	15	11
Categorical and continuous	12	6	2	4
Sleep-measure reporting				
Undefined-reproductible	33	6	14	13
Defined	26	5	9	12
Undefined	25	1	13	11
Sub-sample of studies reporting sex (n = 46)				
Categorical	26	4	15	7
Continuous	20	4	9	7

### Risk of bias in studies

Most studies were classified as having low risk of bias (n = 59), while 25 were classified as having moderate risk; none were classified as having high risk. Differences in classification primarily reflected the clarity and reproducibility of the sleep-duration assessment method. Study-level assessments are presented in Supplementary Table S1.

### Prevalence of adequate sleep duration

**Figure 2:**
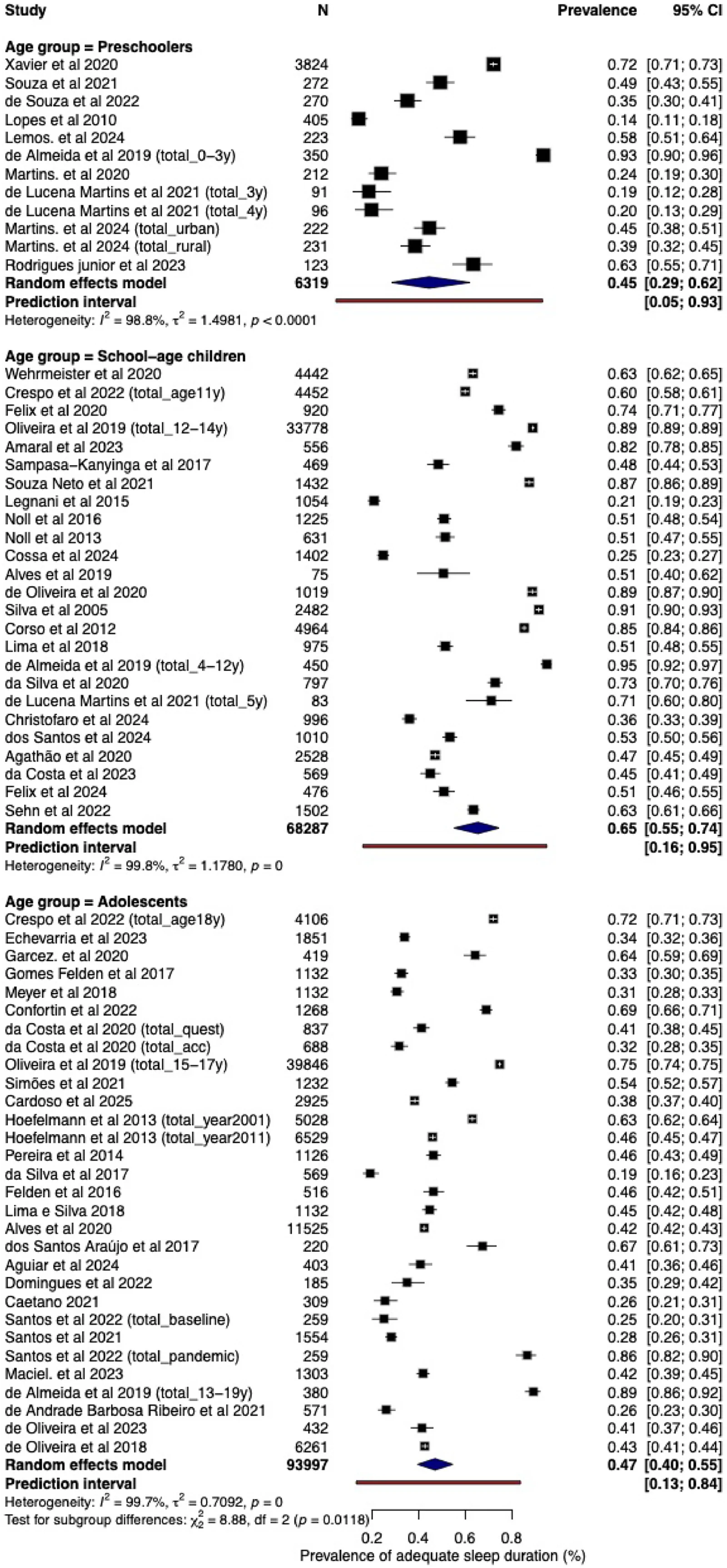
Forest plot of the pooled prevalence estimates by age group.

Fifty-three studies, including 168,603 participants, contributed to the meta-analysis of compliance with sleep-duration recommendations. The pooled prevalence was 44.5% among preschoolers (95% CI: 28.5%–61.8%), 65.4% among school-age children (95% CI: 55.2%–74.4%), and 47.0% among adolescents (95% CI: 39.6%–54.6%). The corresponding 95% prediction intervals were 4.6%–93.0%, 16.1%–94.9%, and 13.4%–83.6%, respectively, indicating substantial between-study variability. Estimates differed significantly across age groups (p = 0.0118; Figure 2).

The size of each square is proportional to the study’s inverse-variance weight in the random-effects model rather than directly to its sample size. Because between-study heterogeneity was substantial, study weights were relatively similar despite differences in sample size.

Questionnaire-based estimates were generally higher than accelerometer-based estimates, although accelerometer data were limited, particularly among preschoolers and school-age children. A subgroup test comparing strata jointly defined by age group and measurement method was statistically significant (p = 0.0294; Supplementary Fig. S1); this was not a formal age-group-vs.-measurement-method interaction test. Among the subset of 26 studies reporting sex-specific prevalence estimates, pooled estimates were similar between boys and girls within age groups (p = 0.8922; Supplementary Fig. S2). Estimates also differed according to the guideline used to classify adequate sleep duration (p < 0.0001), with generally higher estimates under the National Sleep Foundation recommendations and lower estimates under the Canadian guidelines (Supplementary Fig. S3).

### Mean sleep duration

**Figure 3:**
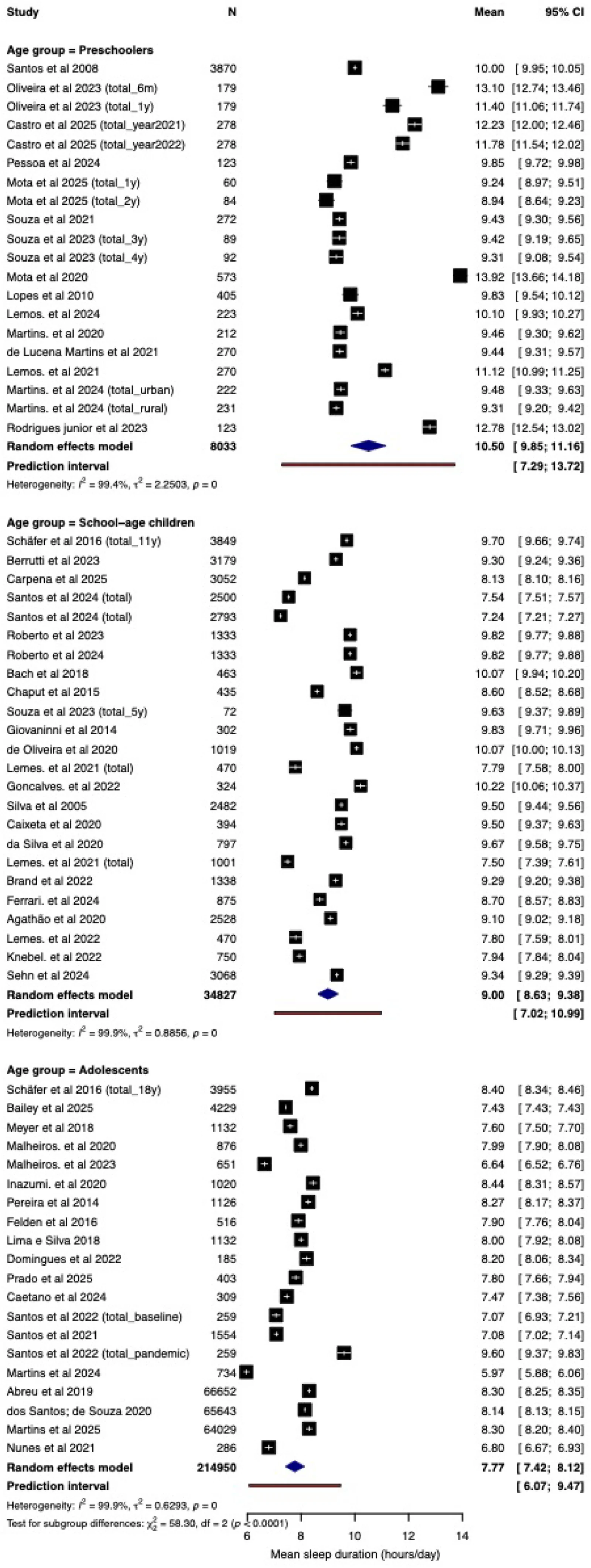
Forest plot of the pooled mean sleep duration by age group.

Forty-three studies contributed to the meta-analysis of mean sleep duration. The pooled mean was 10.50 h/day among preschoolers (95% CI: 9.85–11.20), 9.00 h/day among school-age children (95% CI: 8.63–9.38), and 7.77 h/day among adolescents (95% CI: 7.42–8.12). The corresponding 95% prediction intervals were 7.29–13.70, 7.02–11.00, and 6.07–9.47 h/day, respectively. Mean sleep duration differed significantly across age groups (p < 0.0001; Figure 3).

The size of each square is proportional to the study’s inverse-variance weight in the random-effects model rather than directly to its sample size. Because between-study heterogeneity was substantial, study weights were relatively similar despite differences in sample size.

Among the subset of 20 studies reporting sex-specific mean sleep duration, pooled estimates were similar for boys and girls within each age group (Supplementary Fig. S4). The subgroup test presented in the figure compared all age-by-sex subgroups simultaneously and therefore was not interpreted as a specific test of differences between boys and girls. An omnibus comparison across jointly defined age-group and measurement-method strata was statistically significant (p < 0.0001; Supplementary Fig. S5). Questionnaire-based assessment was associated with higher pooled estimates than accelerometer-based assessment, particularly among school-age children, although accelerometer-based evidence was limited.

### Meta-regression analyses

For the prevalence outcome, there was no evidence that guideline, measurement method, or risk of bias was associated with estimates among school-age children or adolescents, and the multivariable models explained only a small proportion of the between-study heterogeneity. For mean sleep duration among school-age children, questionnaire-based assessment was associated with a longer duration than accelerometer-based assessment in both univariable and multivariable models (adjusted β = 1.23 h; 95% CI: 0.32–2.13; p = 0.0078), with the multivariable model explaining approximately 26.8% of the heterogeneity. Neither measurement method nor risk of bias was significantly associated with mean sleep duration among adolescents. Substantial residual heterogeneity remained across the analyses.

### Reporting bias

Egger’s regression test indicated funnel plot asymmetry for both the prevalence meta-analysis (p = 0.0041) and the mean sleep-duration meta-analysis (p < 0.0001). However, given the substantial between-study heterogeneity and the limitations of asymmetry tests in this context, these findings should be interpreted cautiously. The observed asymmetry may reflect publication bias, heterogeneity-related small-study effects, or a combination of these factors (Supplementary Figs. S6 and S7).

## Discussion

This review presents the first pooled synthesis of sleep duration and compliance with sleep recommendations among Brazilian children and adolescents. The main epidemiological finding is that insufficient sleep duration is common, particularly during adolescence, when approximately half of the population did not meet age-specific recommendations. This finding should not be interpreted solely as an expected consequence of development. Rather, it indicates that a substantial proportion of Brazilian adolescents may lack sufficient opportunities to meet their biological sleep needs.

The decline in sleep duration across age groups aligns with developmental changes in sleep regulation. During adolescence, a delayed circadian phase and a slower build-up of homeostatic sleep pressure favor later sleep onset, while sleep need remains substantial [104]. However, biological maturation alone is unlikely to account for the observed pattern. Early school schedules, academic demands, commuting, employment, social activities, and nighttime technology use may prevent adolescents from compensating for later bedtimes, creating a mismatch between biological timing and the social organization of daily life [105].

The Brazilian findings should also be considered in light of substantial international variation in sleep. In a standardized survey conducted across 24 European and North American countries, the proportion of adolescents meeting sleep recommendations on school days ranged from 32% to 86%, with older adolescents consistently sleeping less than younger ones [106]. The Brazilian pooled estimate therefore falls within the broad international range but suggests a less favourable pattern than that reported in some nationally representative studies, including Canada [10]. Direct comparisons should, however, be made cautiously because international studies differ in age ranges, reference days, measurement instruments, and recommendation criteria.

The wide variation observed across countries suggests that adolescent sleep is shaped not only by biological development but also by the social and environmental organization of daily life [2]. This may be particularly relevant in Brazil, where marked regional and socioeconomic inequalities can influence sleep opportunities through differences in housing conditions, household crowding, neighbourhood noise and safety, exposure to violence, commuting demands, parental work schedules, and access to suitable sleeping environments [107,108]. School organization may also play an important role, as early morning schedules conflict with adolescents’ delayed biological timing, while academic demands and nighttime screen use may further restrict sleep [109]. Urbanization, seasonality, and regional differences in climate and natural light exposure may additionally affect sleep timing, duration, and continuity [110]. Because these characteristics differ substantially across Brazilian populations and between regions, they may contribute to the broad variability observed in this review; however, as they were inconsistently reported across studies, they should be considered plausible contributors rather than established explanations for the heterogeneity.

The subgroup analysis showed that studies using questionnaires reported longer sleep duration and a higher prevalence of meeting recommendations than those using accelerometers. However, heterogeneity in sleep assessment extends beyond instrument type. Sleep duration may refer to nocturnal sleep or total sleep over 24 h, with or without daytime naps; it may be assessed separately for school days and free days or averaged across the week; and questions may refer to habitual sleep, a recent period, time in bed, or estimated time actually asleep. These definitions are not interchangeable and may be particularly relevant for preschoolers, for whom daytime sleep contributes to total sleep duration, and for adolescents, whose sleep often differs substantially between school and non-school days. Self-reported sleep measures are prone to recall and social desirability biases, which can lead to overestimation [1]. In addition, subgroup analysis identified differences in the prevalence of meeting recommendations across guidelines and cut-off points. This issue was noted in the limitations of previous systematic reviews of sleep duration [13,14], and this subgroup analysis highlights the influence of different thresholds on the prevalence of adequate sleep. Therefore, variation across studies may reflect differences in both the underlying sleep construct and the criteria applied to classify compliance. No differences in sleep duration or in the prevalence of meeting recommendations were found between boys and girls in this review. Although previous studies have shown that girls sleep a little longer, these differences are usually small and might not have epidemiological significance. The present findings should nevertheless be interpreted cautiously because sex-stratified analyses were based on only a subset of the included studies.

Despite differences observed across measurement methods and guideline criteria, substantial between-study heterogeneity remained unexplained. The broad age-specific 95% prediction intervals indicate that estimates from a future comparable study may differ considerably from the pooled prevalence or mean sleep duration. Therefore, the pooled estimates should not be interpreted as precise national parameters that apply uniformly to all Brazilian children and adolescents, but rather as central summaries of a highly heterogeneous evidence base. This variability likely reflects a combination of methodological differences and contextual characteristics, including socioeconomic conditions, school schedules, urban environments, regional inequalities, and behavioural factors that were not consistently reported across studies. Although high heterogeneity is common in prevalence meta-analyses [111], its magnitude in the present review reduces the precision and generalizability of the pooled estimates. Several factors explain this finding, including genuine variation in prevalence across populations [112], large sample sizes that can inflate I^2^ [113], and methodological differences across prevalence studies [114]. Study-design differences may also have contributed to the observed variability. However, the evidence was predominantly cross-sectional, with relatively few longitudinal studies, which limited the ability to examine study design as a reliable source of heterogeneity. Accordingly, the persistence of heterogeneity should be considered both an important limitation and an indication of the context-dependent nature of sleep behaviour.

Several limitations should be considered. First, most included studies were cross-sectional, limiting the ability to examine longitudinal changes in sleep duration, temporal trends, or individual developmental trajectories. In addition, some summary statistics and prevalence estimates were derived from the information reported in the original studies and may have been affected by rounding or inconsistencies in the reported denominators. Second, the evidence relied predominantly on self- or proxy-reported sleep, with very few studies using accelerometry. Measurement error, recall bias, and differences in how participants interpreted sleep questions may therefore have affected the pooled estimates. Third, this review focused specifically on sleep duration and compliance with duration recommendations. It does not provide a comprehensive assessment of multidimensional sleep health, including sleep quality or satisfaction, timing, regularity, efficiency, and daytime alertness. Fourth, the geographic distribution of the evidence was markedly uneven. Most studies were conducted in the South, Northeast, and Southeast, only two provided nationwide estimates, and the North and Central-West regions were not adequately represented. Consequently, the pooled estimates should not be interpreted as uniformly applicable across all Brazilian regions. Fifth, some studies collected data during or overlapping with the COVID-19 pandemic, when school closures, remote education, social restrictions, and changes in daily routines may have affected sleep. However, data-collection periods were incompletely reported, and the number of studies within pandemic-related categories was insufficient and highly unbalanced, preventing a reliable assessment of this effect. Sixth, heterogeneity remained extremely high after age stratification, subgroup analyses, and meta-regression. The broad prediction intervals indicate that results from future comparable studies may differ substantially from the pooled estimates, and most of the between-study variability remained unexplained. The pooled values should therefore be interpreted as central summaries of a heterogeneous evidence base rather than precise national parameters. Finally, the restrictive eligibility criteria reduced the risk-of-bias assessment's discriminatory capacity. Because all included studies met predefined requirements for representativeness and sample-size adequacy, none could be classified as having high risk of bias, and the distinction between moderate and low risk primarily reflected the quality and reporting of sleep-duration measurement. Additionally, the inclusion of studies with moderate risk of bias may increase uncertainty in the pooled estimates.

Nevertheless, this study demonstrates several notable strengths. First, it updates prior evidence by incorporating recently published studies. It advances the field by applying meta-analytic techniques to quantitatively synthesize sleep duration among Brazilian children and adolescents. Second, including a large number of studies and participants increases the robustness and generalizability of the findings. Third, the emphasis on population-based studies with probabilistic sampling enhances the representativeness of the results. Furthermore, the application of comprehensive analytical approaches, such as subgroup and meta-regression analyses, enables exploration of potential sources of variability. Notably, this study extends beyond descriptive estimates by evaluating whether methodological factors, including measurement instruments and sleep guidelines, are associated with variation in reported sleep outcomes, thereby contributing to a more nuanced interpretation of the existing literature.

The central public health message of this review is that insufficient sleep is widespread among Brazilian adolescents. Nearly half did not meet age-specific sleep recommendations, indicating that inadequate sleep should not be treated only as an individual lifestyle choice. Sleep opportunity is shaped by the interaction between adolescent biology and the organization of family, school, digital, and community life. Accordingly, public health responses should extend beyond individual sleep-hygiene advice and consider school schedules, nighttime technology use, family routines, commuting demands, and the social and environmental conditions in which children and adolescents sleep. Methodological standardization remains important, but primarily because reliable surveillance is necessary to identify inequalities, compare regions and time periods, and evaluate policies. Comparisons across studies, regions, and time periods should be interpreted cautiously unless sleep definitions, reference periods, recommendation criteria, and measurement methods are sufficiently comparable. This is particularly relevant for future Brazilian Report Cards, as apparent differences between surveillance cycles or population groups may partly reflect methodological changes rather than genuine changes in sleep duration. Future Brazilian studies should prioritize clearly defined, reproducible, and consistently reported sleep measures, including explicit reporting of the sleep period assessed and the treatment of school and non-school days. Validated questionnaires remain valuable for large-scale surveillance, while objective methods should be encouraged where feasible to complement self-reported information rather than being regarded as a universally preferable replacement. Geographic coverage should also be expanded to underrepresented regions and populations. Together, these actions would make it possible not only to estimate the magnitude of insufficient sleep more accurately but also to identify which groups face the greatest barriers to obtaining adequate sleep [21].

## Conclusions

This systematic review and meta-analysis synthesized evidence on sleep duration among Brazilian children and adolescents. The results demonstrate a high prevalence of insufficient sleep duration, particularly among adolescents. However, the pooled estimates varied widely across studies and should be interpreted cautiously given the broad prediction intervals and substantial unexplained heterogeneity. Differences were observed across measurement approaches and recommendation criteria, but the methodological moderators evaluated in the meta-regression explained only a limited proportion of the between-study variability, suggesting that additional contextual and population-level factors likely contribute. Future surveillance, including Brazilian Report Card initiatives, should prioritize standardized, transparently reported definitions of sleep, reference periods, and assessment methods, while incorporating objective measurements where feasible. From a public health perspective, strategies should address not only individual sleep behaviours but also the school, family, social, and environmental conditions that shape opportunities for adequate sleep.

## Ethics approval statement

Ethical approval was not required for this study, as it is based on previously published data and does not involve direct human participants.

## Permission to reproduce material from other sources

No material subject to copyright restrictions was used in this study.

## Funding

This research did not receive any specific grant from funding agencies in the public, commercial, or not-for-profit sectors.

## Conflicts of interest

The authors declare no conflicts of interest.

## Data Availability

All data analyzed in this study are derived from previously published studies and are included in the article and its supplementary materials.

## Appendix Supplementary materials

Image, application 1

## Footnotes

Supplementary material associated with this article can be found in the online version at doi:10.1016/j.jped.2026.101608.
