# Supplementary material for: Sleep duration and compliance with sleep recommendations among Brazilian children and adolescents: an updated systematic review and meta-analysis
Source: J Pediatr (Rio J). 2026 Sep 16;102(6):101608. doi: 10.1016/j.jped.2026.101608 (PMC13597199; doi:10.1016/j.jped.2026.101608)
Supplement: Supplementary file 1 [file mmc1.docx]

JPED-D-26-00113

Supplementary Material

# 1. SEARCH STRATEGIES

Pubmed

| ID | Group | Keywords |
| --- | --- | --- |
| #1 | Concept | sleep[MeSH Terms] OR "sleep hygiene"[MeSH Terms] OR "sleep deprivation"[MeSH Terms] OR “Sleep Wake Disorders”[MeSH Terms] OR "Sleep Apnea Syndromes"[MeSH Terms] OR sleep* OR "sleeping habits" OR "sleep loss" OR "sleep time" OR "sleep duration" OR "sleep quantity" OR "time spent asleep" OR "time spent sleeping" OR "time sleeping" OR "time asleep" OR "sleep length" OR "Total sleep" OR “sleep restriction” OR "sleep lack" OR "insufficient sleep" OR "poor sleep" OR "short sleep" OR "inadequate sleep" OR "sleep debt" OR "sleep problem" OR insomnia |
| #2 | Context | Brazil* OR Brazilian |
| #3 | Population | Adolescent[MeSH Terms] OR Child[MeSH Terms] OR youth OR teen* OR adolescent* OR adolescence OR student* OR child* OR "young people" OR toddler |
| #4 | Instruments | **Smartphone[MeSH Terms] OR Polysomnography[MeSH Terms] OR "Self Report"[MeSH Terms] OR "Surveys and Questionnaires"[MeSH Terms] OR actigraph* OR "smart watch" OR smartwatch OR wearable OR armband* OR “arm band” OR smartphone OR polysomnography OR polysomnogr* OR "Pittsburgh Sleep Quality Index" OR report OR questionnaire OR diary OR diaries OR log OR self-report OR "self report" OR scale* OR index* OR proxy-report OR checklist* OR recall* OR interview* OR assessment OR survey OR measurement OR acceleromet* OR inclinomet* OR (objectiv* AND measur*) OR (direct* AND measure*)** |

| Syntax | Results |
| --- | --- |
| #1 AND #2 AND #3 AND #4 | 1,068 |

PsychINFO

| ID | Group | Keywords |
| --- | --- | --- |
| #1 | Concept | (((MeSH: (sleep)) OR (MeSH: ("sleep hygiene")) OR (MeSH: ("sleep deprivation")) OR (MeSH: ("Sleep Wake Disorders")) OR (MeSH: ("Sleep Apnea Syndromes"))) AND ((Year: [2020 TO 2026]))) OR (((Any Field: (sleep*) OR Any Field: ("sleeping habits") OR Any Field: ("sleep loss") OR Any Field: ("sleep time") OR Any Field: ("sleep duration") OR Any Field: ("sleep quantity") OR Any Field: ("time spent asleep") OR Any Field: ("time spent sleeping") OR Any Field: ("time sleeping") OR Any Field: ("time asleep") OR Any Field: ("sleep length") OR Any Field: ("Total sleep") OR Any Field: ("sleep restriction") OR Any Field: ("sleep lack") OR Any Field: ("insufficient sleep") OR Any Field: ("poor sleep") OR Any Field: ("short sleep") OR Any Field: ("inadequate sleep") OR Any Field: ("sleep debt") OR Any Field: ("sleep problem") OR Any Field: (insomnia))) AND ((Year: [2020 TO 2026]))) |
| #2 | Context | Any Field: Brazil* OR Any Field: Brazilian AND Year: 2020 TO 2026 |
| #3 | Population | (((MeSH: (Adolescent)) OR (MeSH: (Child))) AND ((Year: [2020 TO 2026]))) OR (((Any Field: (youth) OR Any Field: (teen*) OR Any Field: (adolescent*) OR Any Field: (adolescence) OR Any Field: (student*) OR Any Field: (child*) OR Any Field: ("young people") OR Any Field: (toddler))) AND ((Year: [2020 TO 2026]))) |
| #4 | Instruments | **(((Any Field: (Smartphone)) OR (Any Field: (Polysomnography)) OR (Any Field: ("Self Report")) OR (Any Field: ("Surveys and Questionnaires"))) AND ((Year: [2020 TO 2026]))) OR (((Any Field: (actigraph*) OR Any Field: ("smart watch") OR Any Field: (smartwatch) OR Any Field: (wearable) OR Any Field: (armband*) OR Any Field: ("arm band") OR Any Field: (smartphone) OR Any Field: (polysomnography) OR Any Field: (polysomnogr*) OR Any Field: ("Pittsburgh Sleep Quality Index") OR Any Field: (report) OR Any Field: (questionnaire) OR Any Field: (diary) OR Any Field: (diaries) OR Any Field: (log) OR Any Field: (self-report) OR Any Field: ("self report") OR Any Field: (scale*) OR Any Field: (index*) OR Any Field: (proxy-report) OR Any Field: (checklist*) OR Any Field: (recall*) OR Any Field: (interview*) OR Any Field: (assessment) OR Any Field: (survey) OR Any Field: (measurement) OR Any Field: (acceleromet*) OR Any Field: (inclinomet*) OR (Any Field: (objectiv*) AND Any Field: (measur*)) OR (Any Field: (direct*) AND Any Field: (measure*)))) AND ((Year: [2020 TO 2026])))** |

| Syntax | Results |
| --- | --- |
| #1 AND #2 AND #3 AND #4 | 241 |

CINAHL

| ID | Group | Keywords |
| --- | --- | --- |
| #1 | Concept | TI ( sleep* OR "sleeping habits" OR "sleep hygiene" OR "sleep deprivation" OR "sleep loss" OR "sleep time" OR "sleep duration" OR "sleep quantity" OR "time spent asleep" OR "time spent sleeping" OR "time sleeping" OR "time asleep" OR "sleep length" OR "Total sleep" OR “sleep restriction” OR "sleep lack" OR "insufficient sleep" OR "poor sleep" OR "short sleep" OR "inadequate sleep" OR "sleep debt" OR "sleep problem" OR "Sleep Apnea" OR insomnia ) OR AB ( sleep* OR "sleeping habits" OR "sleep hygiene" OR "sleep deprivation" OR "sleep loss" OR "sleep time" OR "sleep duration" OR "sleep quantity" OR "time spent asleep" OR "time spent sleeping" OR "time sleeping" OR "time asleep" OR "sleep length" OR "Total sleep" OR “sleep restriction” OR "sleep lack" OR "insufficient sleep" OR "poor sleep" OR "short sleep" OR "inadequate sleep" OR "sleep debt" OR "sleep problem" OR "Sleep Apnea" OR insomnia ) OR MW ( sleep* OR "sleeping habits" OR "sleep hygiene" OR "sleep deprivation" OR "sleep loss" OR "sleep time" OR "sleep duration" OR "sleep quantity" OR "time spent asleep" OR "time spent sleeping" OR "time sleeping" OR "time asleep" OR "sleep length" OR "Total sleep" OR “sleep restriction” OR "sleep lack" OR "insufficient sleep" OR "poor sleep" OR "short sleep" OR "inadequate sleep" OR "sleep debt" OR "sleep problem" OR "Sleep Apnea" OR insomnia ) OR SU ( sleep* OR "sleeping habits" OR "sleep hygiene" OR "sleep deprivation" OR "sleep loss" OR "sleep time" OR "sleep duration" OR "sleep quantity" OR "time spent asleep" OR "time spent sleeping" OR "time sleeping" OR "time asleep" OR "sleep length" OR "Total sleep" OR “sleep restriction” OR "sleep lack" OR "insufficient sleep" OR "poor sleep" OR "short sleep" OR "inadequate sleep" OR "sleep debt" OR "sleep problem" OR "Sleep Apnea" OR insomnia ) |
| #2 | Context | TI ( Brazil* OR Brazilian ) OR AB ( Brazil* OR Brazilian ) OR MW ( Brazil* OR Brazilian ) OR SU ( Brazil* OR Brazilian ) OR AF ( Brazil* OR Brazilian ) |
| #3 | Population | TI ( youth OR teen* OR adolescent* OR adolescence OR student* OR child* OR “young people” OR toddler ) OR AB ( youth OR teen* OR adolescent* OR adolescence OR student* OR child* OR “young people” OR toddler ) OR MW ( youth OR teen* OR adolescent* OR adolescence OR student* OR child* OR “young people” OR toddler ) OR SU ( youth OR teen* OR adolescent* OR adolescence OR student* OR child* OR “young people” OR toddler ) |
| #4 | Instruments | TI ( actigraph* OR "smart watch" OR smartwatch OR wearable OR armband* OR “arm band” OR smartphone OR polysomnography OR polysomnogr* OR "Pittsburgh Sleep Quality Index" OR report OR questionnaire OR diary OR diaries OR log OR self-report OR "self report" OR scale* OR index* OR proxy-report OR checklist* OR recall* OR interview* OR assessment OR survey OR measurement OR acceleromet* OR inclinomet* OR (objectiv* AND measur*) OR (direct* AND measure*) ) OR AB (actigraph* OR "smart watch" OR smartwatch OR wearable OR armband* OR “arm band” OR smartphone OR polysomnography OR polysomnogr* OR "Pittsburgh Sleep Quality Index" OR report OR questionnaire OR diary OR diaries OR log OR self-report OR "self report" OR scale* OR index* OR proxy-report OR checklist* OR recall* OR interview* OR assessment OR survey OR measurement OR acceleromet* OR inclinomet* OR (objectiv* AND measur*) OR (direct* AND measure*) ) OR MW (actigraph* OR "smart watch" OR smartwatch OR wearable OR armband* OR “arm band” OR smartphone OR polysomnography OR polysomnogr* OR "Pittsburgh Sleep Quality Index" OR report OR questionnaire OR diary OR diaries OR log OR self-report OR "self report" OR scale* OR index* OR proxy-report OR checklist* OR recall* OR interview* OR assessment OR survey OR measurement OR acceleromet* OR inclinomet* OR (objectiv* AND measur*) OR (direct* AND measure*) ) OR SU (actigraph* OR "smart watch" OR smartwatch OR wearable OR armband* OR “arm band” OR smartphone OR polysomnography OR polysomnogr* OR "Pittsburgh Sleep Quality Index" OR report OR questionnaire OR diary OR diaries OR log OR self-report OR "self report" OR scale* OR index* OR proxy-report OR checklist* OR recall* OR interview* OR assessment OR survey OR measurement OR acceleromet* OR inclinomet* OR (objectiv* AND measur*) OR (direct* AND measure*) ) |

| Syntax | Results |
| --- | --- |
| #1 AND #2 AND #3 AND #4 | 293 |

SCIELO

| ID | Group | Keywords |
| --- | --- | --- |
| #1 | Concept | sleep* OR insomnia OR sono OR **dormir OR insônia** |
| #2 | Context | Brazil* OR Brasil* |
| #3 | Population | youth OR teen* OR adolescent* OR adolescence OR student* OR child* OR “young people” OR toddler OR jovem OR adolescente* OR adolescência OR estudante* OR criança* OR jovens OR infância OR bebês OR aluno* OR juventude |
| #4 | Instruments | **actigraph* OR "smart watch" OR smartwatch OR wearable OR armband* OR “arm band” OR smartphone OR polysomnogr* OR "Pittsburgh Sleep Quality Index" OR questionnaire OR diary OR diaries OR log OR self-report OR "self report" OR scale OR index OR proxy-report OR checklist OR recall OR interview* OR survey OR acceleromet* OR inclinomet* OR “objective measure” OR “objectively measured” OR polissonografia OR polissonógrafo* OR “Índice de Qualidade do Sono de Pittsburgh” OR relatório OR questionário OR diário* OR registro OR escala OR índice OR proxy-report OR checklist OR recordatório OR entrevistas OR acelerômetr* OR inclinômet* OR “medida objetiva” OR auto-relato OR avalia* OR mensura* OR inspecionar** |

| Syntax | Results |
| --- | --- |
| #1 AND #2 AND #3 AND #4 | 114 |

EMBASE

| ID | Group | Keywords |
| --- | --- | --- |
| #1 | Concept | sleep* OR "sleeping habits" OR "sleep hygiene" OR "sleep deprivation" OR "sleep loss" OR "sleep time" OR "sleep duration" OR "sleep quantity" OR "time spent asleep" OR "time spent sleeping" OR "time sleeping" OR "time asleep" OR "sleep length" OR "Total sleep" OR "sleep lack" OR "insufficient sleep" OR "poor sleep" OR "short sleep" OR "inadequate sleep" OR "sleep debt" OR "sleep problem" OR “sleep apnea" OR insomnia OR “sleep restriction” |
| #2 | Context | Brazil* OR Brazilian |
| #3 | Population | youth OR teen* OR adolescent* OR adolescence OR student* OR child* OR “young people” OR toddler |
| #4 | Instruments | **actigraph* OR "smart watch" OR smartwatch OR wearable OR armband* OR “arm band” OR smartphone OR polysomnography OR polysomnogr* OR "Pittsburgh Sleep Quality Index" OR report OR questionnaire OR diary OR diaries OR log OR self-report OR "self report" OR scale* OR index* OR proxy-report OR checklist* OR recall* OR interview* OR assessment OR survey OR measurement OR acceleromet* OR inclinomet* OR (objectiv* AND measur*) OR (direct* AND measure*)** |
| #5 | Filtros | AND [2020-2026]/py |

| Syntax | Results |
| --- | --- |
| #1 AND #2 AND #3 AND #4 | 1,558 |

Web of Science

| ID | Group | Keywords |
| --- | --- | --- |
| #1 | Concept | ALL=(sleep* OR "sleeping habits" OR "sleep hygiene" OR "sleep deprivation" OR "sleep loss" OR "sleep time" OR "sleep duration" OR "sleep quantity" OR "time spent asleep" OR "time spent sleeping" OR "time sleeping" OR "time asleep" OR "sleep length" OR "Total sleep" OR "sleep lack" OR "insufficient sleep" OR "poor sleep" OR "short sleep" OR "inadequate sleep" OR "sleep debt" OR "sleep problem" OR “sleep apnea" OR insomnia OR “sleep restriction”) |
| #2 | Context | ALL=(Brazil* OR Brazilian) |
| #3 | Population | ALL=(youth OR teen* OR adolescent* OR adolescence OR student* OR child* OR “young people” OR toddler) |
| #4 | Instruments | **ALL=( actigraph* OR "smart watch" OR smartwatch OR wearable OR armband* OR “arm band” OR smartphone OR polysomnography OR polysomnogr* OR "Pittsburgh Sleep Quality Index" OR report OR questionnaire OR diary OR diaries OR log OR self-report OR "self report" OR scale* OR index* OR proxy-report OR checklist* OR recall* OR interview* OR assessment OR survey OR measurement OR acceleromet* OR inclinomet* OR (objectiv* AND measur*) OR (direct* AND measure*))** |
| #5 | Filtros | **Tempo estipulado=2020-2025** |

| Syntax | Results |
| --- | --- |
| #1 AND #2 AND #3 AND #4 | 934 |

Scopus

| ID | Group | Keywords |
| --- | --- | --- |
| #1 | Concept | TITLE-ABS-KEY(sleep* OR "sleeping habits" OR "sleep hygiene" OR "sleep deprivation" OR "sleep loss" OR "sleep time" OR "sleep duration" OR "sleep quantity" OR "time spent asleep" OR "time spent sleeping" OR "time sleeping" OR "time asleep" OR "sleep length" OR "Total sleep" OR "sleep lack" OR "insufficient sleep" OR "poor sleep" OR "short sleep" OR "inadequate sleep" OR "sleep debt" OR "sleep problem" OR "sleep apnea" OR insomnia OR "sleep restriction") |
| #2 | Context | (TITLE-ABS-KEY-AUTH(brazil* OR brazilian) OR AFFILCOUNTRY( brazil* OR brazilian )) |
| #3 | Population | TITLE-ABS-KEY-AUTH(youth OR teen* OR adolescent* OR adolescence OR student* OR child* OR "young people" OR toddler) |
| #4 | Instruments | TITLE-ABS-KEY-AUTH(actigraph* OR "smart watch" OR smartwatch OR wearable OR armband* OR "arm band" OR smartphone OR polysomnography OR polysomnogr* OR "Pittsburgh Sleep Quality Index" OR report OR questionnaire OR diary OR diaries OR log OR self-report OR "self report" OR scale* OR index* OR proxy-report OR checklist* OR recall* OR interview* OR assessment OR survey OR measurement OR acceleromet* OR inclinomet* OR (objectiv* AND measur*) OR (direct* AND measure*)) |
| #5 | Filtros | AND PUBYEAR > 2019 AND PUBYEAR < 2027 |

| Syntax | Results |
| --- | --- |
| #1 AND #2 AND #3 AND #4 | 1236 |

LILACS

| ID | Group | Keywords |
| --- | --- | --- |
| #1 | Concept | ((tw:(sleep*)) OR (tw:(insomnia)) OR (tw:(sono)) OR (tw:(**dormir)) OR (tw:(insônia)))** |
| #2 | Context | ((tw:(Brazil*)) OR (tw:(Brasil*))) |
| #3 | Population | ((tw:(youth)) OR (tw:(teen*)) OR (tw:(adolescent*)) OR (tw:(adolescence)) OR (tw:(student*)) OR (tw:(child*)) OR (tw:(“young people”)) OR (tw:(toddler)) OR (tw:(jovem)) OR (tw:(adolescente*)) OR (tw:(adolescência)) OR (tw:(estudante*)) OR (tw:(criança*)) OR (tw:(jovens)) OR (tw:(infância)) OR (tw:(bebê*)) OR (tw:(jovem))) |
| #4 | Instruments | ((tw:(**actigraph*)) OR (tw:("smart watch")) OR (tw:(smartwatch)) OR (tw:(wearable)) OR (tw:(armband*)) OR (tw:(“arm band”)) OR (tw:(smartphone)) OR (tw:(polysomnogr*)) OR (tw:("Pittsburgh Sleep Quality Index")) OR (tw:(questionnaire)) OR (tw:(diary)) OR (tw:(diaries)) OR (tw:(log)) OR (tw:(self-report)) OR (tw:("self report")) OR (tw:(scale*)) OR (tw:(index*)) OR (tw:(proxy-report)) OR (tw:(checklist*)) OR (tw:(recall*)) OR (tw:(interview*)) OR (tw:(survey)) OR (tw:(acceleromet*)) OR (tw:(inclinomet*)) OR (tw:(“objective measure”)) OR (tw:(“objectively measured”)) OR (tw:(polissonografia)) OR (tw:(polissonógrafo*)) OR (tw:(relatório)) OR (tw:(questionário)) OR (tw:(diário*)) OR (tw:(registro*)) OR (tw:(escala*)) OR (tw:(índice*)) OR (tw:(proxy-report)) OR (tw:(recordatório*)) OR (tw:(entrevista*)) OR (tw:(acelerômetr*)) OR (tw:(inclinômet*)) OR (tw:(“medida objetiva”)))** |
| #5 | Filtros | AND (year_cluster:[2020 TO 2026]) |

| Syntax | Results |
| --- | --- |
| #1 AND #2 AND #3 AND #4 | 196 |

# 2. Supplementary Table S1 - General characteristics of included studies

Table 1. General characteristics of included studies

| Study label | Study name | Region | Study design | Sample size | Instrument type | Instrument name | Instrument defined | Outcome type | Risk of bias |  |
| --- | --- | --- | --- | --- | --- | --- | --- | --- | --- | --- |
| School-age Children | | | | | | | | | | |
| Wehrmeister et al 2020 | 1993 Pelotas Birth Cohort Study | South | Cross-sectional | 4,442 | Questionnaire | NA | Undefined-reproductible | Categorical only | Low |  |
| Santos et al 2024 | 2015 Pelotas Birth Cohort | South | Longitudinal | 2,793 | Accelerometer | Actigraph-GT3x® | Defined | Continuous only | Low |  |
| Felix et al 2020 | PreK | Southeast | Cross-sectional | 920 | Questionnaire | NA | Undefined | Categorical only | Moderate |  |
| Silva et al 2020 | NA | Northeast | Cross-sectional | 120 | Questionnaire | NA | Undefined-reproductible | Continuous only | Low |  |
| Roberto et al 2023 | EPOCA | South | Cross-sectional | 1,333 | Questionnaire | SSHS | Defined | Not identified | Low |  |
| Amaral et al 2023 | Healthy Childhood in Context | South | Cross-sectional | 556 | Questionnaire | NA | Undefined-reproductible | Categorical only | Low |  |
| Guedes et al 2018 | Healthy Paraná Project | South | Cross-sectional | 8,776 | Questionnaire | NA | Undefined-reproductible | Categorical only | Low |  |
| Chaput et al 2015 | ISCOLE | Southeast | Cross-sectional | 435 | Accelerometer | ActiGraph GT3X+ | Defined | Continuous only | Low |  |
| Barreto et al 2025 | LONCAAFS Study | Northeast | Longitudinal | 544 | Questionnaire | NA | Undefined-reproductible | Continuous only | Low |  |
| Benedet et al 2013 | NA | South | Cross-sectional | 734 | Questionnaire | NA | Undefined | Categorical only | Moderate |  |
| Brandalize 2011 | NA | South | Longitudinal | 212 | Questionnaire | NA | Undefined-reproductible | Continuous only | Low |  |
| Noll et al 2013 | NA | South | Cross-sectional | 1,337 | Questionnaire | BackPEI | Defined | Categorical only | Low |  |
| Legnani et al 2015 | NA | South | Cross-sectional | 1,054 | Questionnaire | NA | Undefined | Categorical only | Moderate |  |
| Noll et al 2016 | NA | South | Cross-sectional | 1,225 | Questionnaire | BackPEI | Defined | Categorical only | Low |  |
| Giovaninni et al 2014 | NA | Southeast | Cross-sectional | 302 | Questionnaire | NA | Undefined | Not identified | Moderate |  |
| Alves et al 2019 | NA | Northeast | Cross-sectional | 75 | Questionnaire | NA | Undefined | Categorical only | Moderate |  |
| Prado et al 2023 | NA | Southeast | Cross-sectional | 172 | Questionnaire | SBQ | Defined | Categorical only | Low |  |
| de Oliveira et al 2020 | NA | South | Cross-sectional | 1,019 | Questionnaire | SSHS | Defined | Categorical only | Low |  |
| Lemes. et al 2021 | NA | South | Cross-sectional | 470 | Questionnaire | NA | undefined-reproductable | Continuous only | Low |  |
| Goncalves. et al 2022 | NA | Northeast | Cross-sectional | 324 | Questionnaire | NA | undefined | Continuous only | Moderate |  |
| Silva et al 2005 | NA | Southeast | Cross-sectional | 2,482 | Questionnaire | NA | Undefined | Categorical and continuous | Moderate |  |
| Corso et al 2012 | NA | South | Cross-sectional | 4,964 | Questionnaire | NA | Undefined | Categorical only | Moderate |  |
| Lima et al 2018 | NA | South | Cross-sectional | 975 | Questionnaire | NA | Undefined-reproductible | Categorical only | Low |  |
| Caixeta et al 2020 | NA | Southeast | Cross-sectional | 394 | Questionnaire | NA | Undefined | Continuous only | Moderate |  |
| da Silva et al 2020 | NA | Southeast | Cross-sectional | 797 | Questionnaire | NA | Undefined | Categorical only | Moderate |  |
| Oliveira et al 2020 | NA | Southeast | Cross-sectional | 339 | Questionnaire | MCTQ | Defined | Continuous only | Low |  |
| Lemes. et al 2021 | NA | Southeast | Cross-sectional | 1,001 | Questionnaire | NA | undefined | Continuous only | Moderate |  |
| Brand et al 2022 | NA | South | Cross-sectional | 1,338 | Questionnaire | NA | Undefined-reproductible | Continuous only | Low |  |
| Christofaro et al 2024 | NA | Southeast | Cross-sectional | 996 | Questionnaire | NA | Undefined | Categorical only | Moderate |  |
| dos Santos et al 2024 | NA | Southeast | Cross-sectional | 1,010 | Questionnaire | NA | Undefined | Categorical only | Moderate |  |
| Ferrari. et al 2024 | NA | NA | Cross-sectional | 875 | Questionnaire | NA | undefined-reproductable | Continuous only | Low |  |
| Agathão et al 2020 | PAAPAS | Southeast | Longitudinal | 2,528 | Questionnaire | NA | Undefined-reproductible | Categorical and continuous | Low |  |
| Lemes. et al 2022 | PROESP-BR | South | Cross-sectional | 470 | Questionnaire | MBQ | defined | Continuous only | Low |  |
| Costa et al 2021 | Movimente study | South | Cross-sectional | 561 | Questionnaire | NA | Undefined-reproductible | Categorical only | Low |  |
| Felix et al 2024 | School-Health - Phase III | South | Cross-sectional | 476 | Questionnaire | NA | Undefined-reproductible | Categorical only | Low |  |
| Sehn et al 2020 | School1Children’s Health | South | Cross-sectional | 1,411 | Questionnaire | NA | Undefined-reproductible | Categorical only | Low |  |
| Adolescents | | | | | | | | | | |
| Santana et al 2022 | 1997/1998 São Luís Birth Cohort | Northeast | Cross-sectional | 569 | Accelerometer | Actigraph wGT3X-BT | Defined | Continuous only | Low |  |
| Garcez. et al 2020 | 2015 ISA-Nutrition | Southeast | Cross-sectional | 419 | Questionnaire | NA | undefined-reproductable | Categorical only | Low |  |
| Gomes Felden et al 2017 | BRACAH | South | Cross-sectional | 1,132 | Questionnaire | NA | Undefined | Categorical only | Moderate |  |
| Collese 2019 | BRACAH | South | Cross-sectional | 370 | Questionnaire | NA | Undefined-reproductible | Continuous only | Low |  |
| Meyer et al 2018 | Brazilian Guide for Assessment of Physical Fitness Related to Health and Life Habits | South | Cross-sectional | 1,132 | Questionnaire | NA | Undefined | Categorical and continuous | Moderate |  |
| Confortin et al 2022 | Determinants throughout the life cycle of obesity, precursors of chronic diseases, human capital and mental health | Northeast | Longitudinal | 1,268 | Accelerometer | ActiGraph GT3X, GTX3+ | Defined | Categorical only | Low |  |
| da Costa et al 2020 | ELEVA | South | Cross-sectional | 837 | Questionnaire | NA | Undefined-reproductible | Categorical only | Low |  |
| Corrêa et al 2021 | ERICA | Country-wide | Cross-sectional | 65,837 | Questionnaire | NA | Undefined | Categorical only | Moderate |  |
| Simões et al 2021 | NA | South | Cross-sectional | 1,232 | Questionnaire | NA | Undefined | Categorical only | Moderate |  |
| Cardoso et al 2025 | RAPS integration | Northeast | Cross-sectional | 2,925 | Questionnaire | NA | Undefined-reproductible | Categorical only | Low |  |
| Inazumi. et al 2020 | Health Survey of the city of Campinas | Southeast | Cross-sectional | 1,020 | Questionnaire | NA | undefined | Continuous only | Moderate |  |
| De Marco et al 2025 | Levels of physical activity, physical fitness and health-related social behavior in adolescents: A secular trend study | South | Cross-sectional | 477 | Questionnaire | NA | Undefined | Categorical only | Moderate |  |
| Hoefelmann et al 2013 | COMPAC | South | Cross-sectional | 5,028 | Questionnaire | COMPAC questionnaire | Defined | Categorical only | Low |  |
| Hoefelmann et al 2013 | COMPAC | South | Cross-sectional | 6,529 | Questionnaire | COMPAC questionnaire | Defined | Categorical only | Low |  |
| Pereira et al 2014 | NA | South | Cross-sectional | 1,126 | Questionnaire | NA | Undefined | Categorical and continuous | Moderate |  |
| Felden et al 2016 | NA | South | Cross-sectional | 516 | Questionnaire | NA | Undefined | Continuous only | Moderate |  |
| da Silva et al 2017 | NA | Northeast | Cross-sectional | 569 | Questionnaire | NA | Undefined-reproductible | Categorical only | Low |  |
| de Lima et al 2019 | NA | South | Cross-sectional | 861 | Questionnaire | NA | Undefined-reproductible | Categorical only | Low |  |
| dos Santos Araújo et al 2017 | NA | Northeast | Cross-sectional | 220 | Questionnaire | NA | Undefined | Categorical only | Moderate |  |
| Alves et al 2020 | NA | Northeast | Cross-sectional | 11,525 | Questionnaire | NA | Undefined-reproductible | Categorical only | Low |  |
| Alves et al 2020 | NA | Northeast | Cross-sectional | 5,310 | Questionnaire | NA | Undefined-reproductible | Categorical only | Low |  |
| Ferrari Junior et al 2019 | NA | South | Cross-sectional | 773 | Questionnaire | NA | Undefined | Continuous only | Moderate |  |
| de Faria et al 2022 | NA | Southeast | Cross-sectional | 217 | Accelerometer | Actigraph Gt3x | Defined | Continuous only | Low |  |
| Aguiar et al 2024 | NA | Southeast | Cross-sectional | 403 | Questionnaire | NA | Undefined-reproductible | Categorical only | Low |  |
| Prado et al 2025 | NA | Southeast | Cross-sectional | 403 | Questionnaire | NA | Undefined-reproductible | Continuous only | Low |  |
| Caetano 2021 | NA | Southeast | Cross-sectional | 309 | Accelerometer | ActiGraph accelerometer (GT3X Model) | Defined | Categorical only | Low |  |
| Santos et al 2021 | NA | South | Cross-sectional | 1,554 | Questionnaire | PSQI | Defined | Categorical and continuous | Low |  |
| Maciel. et al 2023 | NA | Northeast | Cross-sectional | 1,303 | Questionnaire | NA | undefined-reproductable | Categorical only | Low |  |
| Pacifico et al 2024 | NA | South | Cross-sectional | 1,020 | Questionnaire | PSQI | Defined | Categorical only | Low |  |
| de Andrade Barbosa Ribeiro et al 2021 | NA | Northeast | Cross-sectional | 571 | Questionnaire | NA | Undefined-reproductible | Categorical only | Low |  |
| Ferreira. et al 2022 | NA | Northeast | Cross-sectional | 1,207 | Questionnaire | MCTQ | defined | Continuous only | Low |  |
| de Oliveira et al 2023 | NA | Southeast | Cross-sectional | 432 | Questionnaire | NA | Undefined-reproductible | Categorical only | Low |  |
| Martins et al 2024 | NA | Southeast | Cross-sectional | 734 | Questionnaire | MCTQ | Defined | Continuous only | Low |  |
| Tassitano et al 2020 | Physical Activity and Health Risk Behaviors in High School Students of Pernambuco State (Brazil) study | Northeast | Cross-sectional | 5,250 | Questionnaire | GSHS | Defined | Continuous only | Low |  |
| Nunes et al 2021 | NA | Northeast | Cross-sectional | 286 | Questionnaire | NA | Undefined | Continuous only | Moderate |  |
| Santos et al 2022 | NA | South | Longitudinal | 259 | Questionnaire | PSQI | Defined | Categorical and continuous | Low |  |
| Preschoolers | | | | | | | | | | |
| Carpena et al 2020 | 2004 Pelotas Birth Cohort Study | South | Longitudinal | 3,319 | Questionnaire | NA | Undefined-reproductible | Continuous only | Low |  |
| Silva et al 2024 | Araraquara Cohort Study | Southeast | Cross-sectional | 140 | Questionnaire | BISQ | Defined | Not identified | Low |  |
| Oliveira et al 2023 | SAND | Northeast | Longitudinal | 179 | Questionnaire | BISQ | Defined | Continuous only | Low |  |
| Castro et al 2025 | Iracema-Covid | Northeast | Longitudinal | 278 | Questionnaire | BISQ | Defined | Continuous only | Low |  |
| Lopes et al 2010 | NA | South | Cross-sectional | 405 | Questionnaire | NA | Undefined | Categorical and continuous | Moderate |  |
| Lemos. et al 2024 | NA | Northeast | Cross-sectional | 223 | Questionnaire | NA | undefined-reproductable | Categorical and continuous | Low |  |
| de Almeida et al 2019 | NA | Country-wide | Cross-sectional | 350 | Questionnaire | BISQ | Defined | Categorical only | Low |  |
| Martins. et al 2020 | NA | Northeast | Cross-sectional | 212 | Questionnaire | NA | undefined-reproductable | Categorical and continuous | Low |  |
| de Lucena Martins et al 2021 | NA | Northeast | Cross-sectional | 270 | Questionnaire | NA | Undefined-reproductible | Categorical and continuous | Low |  |
| Lemos. et al 2021 | NA | Northeast | Cross-sectional | 270 | Questionnaire | NA | undefined-reproductable | Continuous only | Low |  |
| Martins. et al 2024 | NA | Northeast | Cross-sectional | 222 | Questionnaire | NA | undefined-reproductable | Categorical and continuous | Low |  |
| Rodrigues junior et al 2023 | SAND | Northeast | Cross-sectional | 123 | Questionnaire | BISQ | Defined | Categorical and continuous | Low |  |

BISQ: Brief Infant Sleep Questionnaire; BRACAH: Brazilian Cardiovascular Adolescent Health; COMPAC: Lifestyle and Risk Behaviors of Young Individuals in Santa Catarina, Brazil; ELEVA: Longitudinal Study of Adolescents Lifestyle; ERICA: Study of Cardiovascular Risk Factors in Adolescents; GSHS: Global School-Based Student Health Survey (adapted version); ISCOLE: International Study of Childhood Obesity, Lifestyle and the Environment; LONCAAFS: Longitudinal Study on Sedentary Behavior, Physical Activity, Dietary Habits, and Health of Adolescents; MBQ: Movement Behavior Questionnaire; MCTQ: Munich Chronotype Questionnaire; PAAPAS: Parents, Students, Community Health Agents and Teachers for Healthy Eating; PreK: Brazilian Preschool Mental Health Study; PROESP-BR: Sport Brazil Project; PSQI: Pittsburgh Sleep Quality Index; SAND: Child Health, Food, Nutrition, and Development; SSHS: School Sleep Habits Survey; SBQ: Sleep Behavior Questionnaire; BackPEI: Back Pain and Body Evaluation Instrument.

# 3. Supplementary Table S2 - Characteristics of studies reporting categorical sleep duration outcomes

Table 2. Characteristics of studies reporting categorical sleep duration outcomes

| Study label | Study name | Region | Sample size | Recommendation* | Subgroup | Prevalence (%) |
| --- | --- | --- | --- | --- | --- | --- |
| School-age Children | | | | | | |
| Agathão et al 2020 | PAAPAS | Southeast | 2,528 | AASM 2016 | Total | 47.1 |
| Amaral et al 2023 | Healthy Childhood in Context | South | 556 | AASM 2016 | Total | 81.8 |
| Christofaro et al 2024 | NA | Southeast | 996 | Canadian guideline | Total | 36.0 |
| Cossa et al 2024 | NA | South | 1,402 | Not reported (≥8 h/day) | Total | 24.8 |
| Crespo et al 2022 | 1993 Pelotas Birth Cohort | South | 4,452 | AASM 2016 | Total_age11y | 59.9 |
| da Costa et al 2023 | Movimente study | South | 569 | AASM 2016 | Total | 45.0 |
| da Silva et al 2020 | NA | Southeast | 797 | NSF | Total | 72.8 |
| de Lucena Martins et al 2021 | NA | Northeast | 83 | Canadian guideline | Total_5y | 71.1 |
| de Oliveira et al 2020 | NA | South | 1,019 | NSF | Total | 88.6 |
| dos Santos et al 2024 | NA | Southeast | 1,010 | Not reported (≥8 h/day) | Total | 53.4 |
| Felix et al 2020 | PreK | Southeast | 920 | Not reported (9–11 h/day) | Total | 74.3 |
| Felix et al 2024 | School-Health - Phase III | South | 476 | NSF | Total | 50.8 |
| Sehn et al 2022 | School1Children’s Health | South | 1,502 | NSF | Total | 63.4 |
| Souza Neto et al 2021 | LONCAAFS | Northeast | 1,432 | NSF | Total | 87.4 |
| Wehrmeister et al 2020 | 1993 Pelotas Birth Cohort Study | South | 4,442 | NSF | Total | 63.2 |
| Alves et al 2019 | NA | Northeast | 75 | AASM 2016 | Total | 50.7 |
| Corso et al 2012 | NA | South | 4,964 | Not reported (≥8 h/day) | Total | 85.2 |
| de Almeida et al 2019 | NA | Country-wide | 450 | NSF | Total_4-12y | 94.9 |
| Legnani et al 2015 | NA | South | 1,054 | Not reported (≥10 h/day) | Total | 20.9 |
| Lima et al 2018 | NA | South | 975 | NSF | Total | 51.4 |
| Noll et al 2016 | NA | South | 1,225 | Not reported (8–9 h/day) | Total | 50.9 |
| Noll et al 2013 | NA | South | 631 | Not reported (8–9 h/day) | Total | 51.3 |
| Oliveira et al 2019 | ERICA | Country-wide | 33,778 | NSF | Total_12-14y | 88.9 |
| Sampasa-Kanyinga et al 2017 | ISCOLE | Southeast | 469 | Canadian guideline | Total | 48.4 |
| Silva et al 2005 | NA | Southeast | 2,482 | Not reported (≥8 h/day) | Total | 91.5 |
| Adolescents | | | | | | |
| Aguiar et al 2024 | NA | Southeast | 403 | AASM 2016 | Total | 40.7 |
| Alves et al 2020 | NA | Northeast | 11,525 | AASM 2016 | Total | 42.4 |
| Caetano 2021 | NA | Southeast | 309 | AASM 2016 | Total | 25.6 |
| Cardoso et al 2025 | RAPS integration | Northeast | 2,925 | Not reported (8–10 h/day) | Total | 38.3 |
| Confortin et al 2022 | NA | Northeast | 1,268 | Not reported (≥6 h/day) | Total | 68.9 |
| Crespo et al 2022 | 1993 Pelotas Birth Cohort | South | 4,106 | AASM 2016 | Total_age18y | 72.0 |
| da Costa et al 2020 | ELEVA | South | 837 | Canadian guideline | Total_quest | 41.2 |
| da Costa et al 2020 | ELEVA | South | 688 | Canadian guideline | Total_acc | 31.7 |
| de Andrade Barbosa Ribeiro et al 2021 | NA | Northeast | 571 | Not reported (≥8 h/day) | Total | 26.1 |
| de Oliveira et al 2023 | NA | Southeast | 432 | NSF | Total | 41.4 |
| Domingues et al 2022 | NA | Southeast | 185 | AASM 2016 | Total | 35.1 |
| Echevarria et al 2023 | 2004 Pelotas Birth Cohort | South | 1,851 | Not reported (≥8 h/day) | Total | 34.0 |
| Garcez. et al 2020 | 2015 ISA-Nutrition | Southeast | 419 | AASM 2016 | Total | 64.2 |
| Maciel. et al 2023 | NA | Northeast | 1,303 | Canadian guideline | Total | 41.9 |
| Santos et al 2022 | NA | South | 259 | Not reported (≥8 h/day) | Total_baseline | 25.1 |
| Santos et al 2022 | NA | South | 259 | Not reported (≥8 h/day) | Total_pandemic | 86.5 |
| Santos et al 2021 | NA | South | 1,554 | NSF | Total | 28.2 |
| Simões et al 2021 | Excesso de peso e características do ambiente escolar em estudantes de Curitiba, Paraná | South | 1,232 | Not reported (≥8 h/day) | Total | 54.4 |
| da Silva et al 2017 | NA | Northeast | 569 | Not reported (≥8 h/day) | Total | 19.3 |
| de Almeida et al 2019 | NA | Country-wide | 380 | NSF | Total_13-19y | 89.2 |
| de Oliveira et al 2018 | Prática de atividades físicas e comportamentos de risco à saúde em estudantes do ensino médio no Estado de Pernambuco | Northeast | 6,261 | Not reported (≥8 h/day) | Total | 42.7 |
| dos Santos Araújo et al 2017 | NA | Northeast | 220 | Not reported (≥8 h/day) | Total | 67.3 |
| Felden et al 2016 | NA | South | 516 | Other reference (≥8 h/day) | Total | 46.4 |
| Gomes Felden et al 2017 | A Brazilian Guide for Evaluating Physical Fitness Related to Health and Life Habits | South | 1,132 | NSF | Total | 32.6 |
| Hoefelmann et al 2013 | COMPAC | South | 5,028 | NSF | Total_year2001 | 63.0 |
| Hoefelmann et al 2013 | COMPAC | South | 6,529 | NSF | Total_year2011 | 46.0 |
| Lima e Silva 2018 | NA | South | 1,132 | Not reported (≥8 h/day) | Total | 44.7 |
| Meyer et al 2018 | Brazilian Guide for Assessment of Physica lFitness Related to Health and Life Habits | South | 1,132 | Other reference (≥8.33 h/day) | Total | 30.7 |
| Oliveira et al 2019 | ERICA | Country-wide | 39,846 | NSF | Total_15-17y | 74.7 |
| Pereira et al 2014 | NA | South | 1,126 | Other reference (≥8 h/day) | Total | 46.4 |
| Preescholers | | | | | | |
| de Lucena Martins et al 2021 | NA | Northeast | 91 | Canadian guideline | Total_3y | 18.7 |
| de Lucena Martins et al 2021 | NA | Northeast | 96 | Canadian guideline | Total_4y | 19.8 |
| de Souza et al 2022 | Movement's cool | Northeast | 270 | Canadian guideline | Total | 35.2 |
| Lemos. et al 2024 | NA | Northeast | 223 | Canadian guideline | Total | 57.8 |
| Martins. et al 2020 | NA | Northeast | 212 | Canadian guideline | Total | 24.0 |
| Martins. et al 2024 | NA | Northeast | 222 | Canadian guideline | Total_urban | 44.6 |
| Martins. et al 2024 | NA | Northeast | 231 | Canadian guideline | Total_rural | 38.5 |
| Rodrigues junior et al 2023 | SAND | Northeast | 123 | AASM 2016 | Total | 63.4 |
| Souza et al 2021 | Movement's Cool | Northeast | 272 | AASM 2016 | Total | 49.3 |
| Xavier et al 2020 | 2004 Pelotas Birth Cohort Study | South | 3,824 | NSF | Total | 72.1 |
| de Almeida et al 2019 | NA | Country-wide | 350 | NSF | Total_0-3y | 93.4 |
| Lopes et al 2010 | NA | South | 405 | Other reference (12–12 h/day) | Total | 14.3 |

NSF: National Sleep Foundation; PAAPAS: Parents, Students, Community Health Agents and Teachers for Healthy Eating; PreK: Brazilian Preschool Mental Health Study; LONCAAFS: Longitudinal Study on Sedentary Behavior, Physical Activity, Eating Habits and Adolescent Health; ERICA: Study of Cardiovascular Risks in Adolescents; ISCOLE: International Study of Childhood Obesity, Lifestyle and the Environment; ELEVA: Longitudinal Study of Adolescents Lifestyle; COMPAC: Lifestyle and Risk Behaviors of Young Individuals in Santa Catarina, Brazil; SAND: Health, Food, Nutrition, and Child Development: a Cohort Study.

* AASM: American Academy of Sleep Medicine (2016) — Preschoolers (3–5 years): 10–13 h/night; Children (6–12 years): 9–12 h/night; Adolescents (13–18 years): 8–10 h/night; NSF: National Sleep Foundation — Preschoolers (3–5 years): 10–13 h/night; Children (6–13 years): 9–11 h/night; Adolescents (14–17 years): 8–10 h/night; Canadian guidelines: Canadian 24-Hour Movement Guidelines — Early Years (0–4 years): 10–13 h/24 h, including naps (3–4 years); Children and Youth (5–17 years): 9–11 h/night (5–13 years) and 8–10 h/night (14–17 years).

# 4. Supplementary Table S3 - Characteristics of studies reporting continuous sleep duration outcomes

Table 3. Characteristics of studies reporting continuous sleep duration outcomes

| Study label | Study name | Region | Sample size | Subgroup | Mean (SD) | Instrument type | Instrument name |  |
| --- | --- | --- | --- | --- | --- | --- | --- | --- |
| School-age Children | | | | | | | | |
| Agathão et al 2020 | PAAPAS | Southeast | 2,528 | Total | 9.1 (2.1) | Questionnaire | NA |  |
| Berrutti et al 2023 | 2004 Pelotas Birth Cohort Study | South | 3,179 | Total | 9.3 (1.7) | Questionnaire | PSQI |  |
| Brand et al 2022 | NA | South | 1,338 | Total | 9.29 (1.61) | Questionnaire | NA |  |
| Caixeta et al 2020 | NA | Southeast | 394 | Total | 9.5 (1.3) | Questionnaire | NA |  |
| Carpena et al 2025 | 2004 Pelotas Birth Cohort Study | South | 3,052 | Total | 8.13 (0.83) | Accelerometer | ActiGraph, wGT3X-BT |  |
| da Silva et al 2020 | NA | Southeast | 797 | Total | 9.67 (1.19)* | Questionnaire | NA |  |
| de Oliveira et al 2020 | NA | South | 1,019 | Total | 10.07 (1.04)* | Questionnaire | SSHS |  |
| Ferrari. et al 2024 | NA | NA | 875 | Total | 8.7 (1.9) | Questionnaire | NA |  |
| Goncalves. et al 2022 | NA | Northeast | 324 | Total | 10.22 (1.45) | Questionnaire | NA |  |
| Knebel. et al 2022 | Movimente study | South | 750 | Total | 7.94 (1.44) | Questionnaire | NA |  |
| Lemes. et al 2021 | NA | South | 470 | Total | 7.79 (2.31) | Questionnaire | NA |  |
| Lemes. et al 2021 | NA | Southeast | 1,001 | Total | 7.5 (1.7) | Questionnaire | NA |  |
| Lemes. et al 2022 | PROESP-BR | South | 470 | Total | 7.8 (2.31) | Questionnaire | MBQ |  |
| Roberto et al 2023 | EPOCA | South | 1,333 | Total | 9.82 (1.04)* | Questionnaire | SSHS |  |
| Roberto et al 2024 | EPOCA | South | 1,333 | Total | 9.82 (1.04)* | Questionnaire | SSHS |  |
| Santos et al 2024 | 2015 Pelotas Birth Cohort | South | 2,793 | Total | 7.24 (0.94) | Accelerometer | Actigraph-GT3x® |  |
| Santos et al 2024 | 2004 Pelotas Birth Cohort | South | 2,500 | Total | 7.54 (0.77) | Accelerometer | GENEActiv® |  |
| Sehn et al 2024 | School1Children’s Health | South | 3,068 | Total | 9.34 (1.42) | Questionnaire | NA |  |
| Souza et al 2023 | Movement's Cool | Northeast | 72 | Total_5y | 9.63 (1.13) | Questionnaire | NA |  |
| Bach et al 2018 | Healthy childhood in context: a multidisciplinary research | South | 463 | Total | 10.07 (1.41) | Questionnaire | NA |  |
| Chaput et al 2015 | ISCOLE | Southeast | 435 | Total | 8.6 (0.8) | Accelerometer | ActiGraph GT3X+ |  |
| Giovaninni et al 2014 | NA | Southeast | 302 | Total | 9.83 (1.11)* | Questionnaire | NA |  |
| Schäfer et al 2016 | 1993 Pelotas Birth Cohort | South | 3,849 | Total_11y | 9.7 (1.4) | Questionnaire | NA |  |
| Silva et al 2005 | NA | Southeast | 2,482 | Total | 9.5 (1.59) | Questionnaire | NA |  |
| Adolescents | | | | | | | | |
| Bailey et al 2025 | 2004 Pelotas Birth Cohort Study | South | 4,229 | Total | 7.43 (0.02) | Questionnaire | PSQI |  |
| Caetano et al 2024 | NA | Southeast | 309 | Total | 7.47 (0.81)* | Accelerometer | ActiGraph GT3X |  |
| Domingues et al 2022 | NA | Southeast | 185 | Total | 8.2 (1) | Accelerometer | Actigraph Gt3x |  |
| dos Santos 2020 | ERICA | Country-wide | 65,643 | Total | 8.14 (1.4) | Questionnaire | NA |  |
| Inazumi. et al 2020 | Health Survey of the city of Campinas | Southeast | 1,020 | Total | 8.44 (2.1) | Questionnaire | NA |  |
| Malheiros. et al 2020 | ELEVA | South | 876 | Total | 7.99 (1.3) | Questionnaire | NA |  |
| Malheiros. et al 2023 | ELEVA | South | 651 | Total | 6.64 (1.5) | Accelerometer | GT3X+ and wGT3X+ |  |
| Martins et al 2025 | ERICA | Country-wide | 64,029 | Total | 8.3 (12.91) | Questionnaire | NA |  |
| Martins et al 2024 | NA | Southeast | 734 | Total_weekday_morningshift | 5.97 (1.24) | Questionnaire | MCTQ |  |
| Nunes et al 2021 | Study of postural and painful changes in the cervi cal region associated with the use of smartphones in adolescents | Northeast | 286 | Total | 6.8 (1.1) | Questionnaire | NA |  |
| Prado et al 2025 | NA | Southeast | 403 | Total | 7.8 (1.4) | Questionnaire | NA |  |
| Santos et al 2022 | na | South | 259 | Total_baseline | 7.07 (1.17) | Questionnaire | PSQI |  |
| Santos et al 2022 | na | South | 259 | Total_pandemic | 9.6 (1.92) | Questionnaire | PSQI |  |
| Santos et al 2021 | NA | South | 1,554 | Total | 7.08 (1.25) | Questionnaire | PSQI |  |
| Abreu et al 2019 | ERICA | Country-wide | 66,652 | Total | 8.3 (6.59) | Questionnaire | NA |  |
| Felden et al 2016 | NA | South | 516 | Total | 7.9 (1.6) | Questionnaire | NA |  |
| Lima e Silva 2018 | NA | South | 1,132 | Total | 8 (1.4) | Questionnaire | NA |  |
| Meyer et al 2018 | Brazilian Guide for Assessment of Physical Fitness Related to Health and Life Habits | South | 1,132 | Total | 7.6 (1.7) | Questionnaire | NA |  |
| Pereira et al 2014 | NA | South | 1,126 | Total | 8.27 (1.67) | Questionnaire | NA |  |
| Schäfer et al 2016 | 1993 Pelotas Birth Cohort | South | 3,955 | Total_18y | 8.4 (1.9) | Questionnaire | NA |  |
| Preschoolers | | | | | | | | |
| Castro et al 2025 | Iracema-Covid | Northeast | 278 | Total_year2021 | 12.23 (1.92) | Questionnaire | BISQ |  |
| Castro et al 2025 | Iracema-Covid | Northeast | 278 | Total_year2022 | 11.78 (2.04) | Questionnaire | BISQ |  |
| de Lucena Martins et al 2021 | NA | Northeast | 270 | Total | 9.44 (1.12) | Questionnaire | NA |  |
| Lemos. et al 2024 | NA | Northeast | 223 | Total | 10.1 (1.3) | Questionnaire | NA |  |
| Lemos. et al 2021 | NA | Northeast | 270 | Total | 11.12 (1.11) | Questionnaire | NA |  |
| Martins. et al 2020 | NA | Northeast | 212 | Total | 9.46 (1.2) | Questionnaire | NA |  |
| Martins. et al 2024 | NA | Northeast | 222 | Total_urban | 9.48 (1.11) | Questionnaire | NA |  |
| Martins. et al 2024 | NA | Northeast | 231 | Total_rural | 9.31 (0.84) | Questionnaire | NA |  |
| Mota et al 2025 | Movement's Cool project | Northeast | 60 | Total_1y | 9.24 (1.07) | Questionnaire | NA |  |
| Mota et al 2025 | Movement's Cool project | Northeast | 84 | Total_2y | 8.94 (1.38) | Questionnaire | NA |  |
| Mota et al 2020 | Movement´s cool | Northeast | 573 | Total | 13.92 (3.12) | Questionnaire | NA |  |
| Oliveira et al 2023 | SAND | Northeast | 179 | Total_6m | 13.1 (2.43) | Questionnaire | BISQ |  |
| Oliveira et al 2023 | SAND | Northeast | 179 | Total_1y | 11.4 (2.31) | Questionnaire | BISQ |  |
| Pessoa et al 2024 | Movement's Cool Project | Northeast | 123 | Total | 9.85 (0.75) | Questionnaire | NA |  |
| Rodrigues junior et al 2023 | SAND | Northeast | 123 | Total | 12.78 (1.34) | Questionnaire | BISQ |  |
| Souza et al 2023 | Movement's Cool | Northeast | 89 | Total_3y | 9.42 (1.13) | Questionnaire | NA |  |
| Souza et al 2023 | Movement's Cool | Northeast | 92 | Total_4y | 9.31 (1.12) | Questionnaire | NA |  |
| Lopes et al 2010 | NA | South | 405 | Total | 9.83 (2.95) | Questionnaire | NA |  |
| Santos et al 2008 | 2004 Pelotas Birth Cohort | South | 3,870 | Total | 10 (1.6) | Questionnaire | NA |  |

* Derived from median (IQR)

PAAPAS: Parents, Students, Community Health Agents and Teachers for Healthy Eating; PROESP-BR: Sport Brazil Project; ISCOLE: International Study of Childhood Obesity, Lifestyle and the Environment; ERICA: Study of Cardiovascular Risks in Adolescents; ELEVA: Longitudinal Study of the Lifestyle of Adolescents; SAND: Child Health, Food, Nutrition, and Development — a Cohort Study; PSQI: Pittsburgh Sleep Quality Index; MBQ: Movement Behavior Questionnaire; BISQ: Brief Infant Sleep Questionnaire.

# 5. Supplementary Table S4 - Characteristics of studies reporting categorical sleep duration outcomes for sex

Table 4. Characteristics of studies reporting categorical sleep duration outcomes for sex

| Study label | Study name | Subgroup | Region | Sample size | Recommendation* | Events | Prevalence (%) | Instrument type | Instrument name | Instrument defined |
| --- | --- | --- | --- | --- | --- | --- | --- | --- | --- | --- |
| School-age Children | | | | | | | | | | |
| Agathão et al 2020 | PAAPAS | Boys | Southeast | 1,328 | AASM 2016 | 613 | 46.2 | Questionnaire | NA | Undefined-reproductible |
| Agathão et al 2020 | PAAPAS | Girls | Southeast | 1,200 | AASM 2016 | 577 | 48.1 | Questionnaire | NA | Undefined-reproductible |
| Oliveira dos Santos et al 2021 | LONCAAFS | Girls | Northeast | 748 | NSF | 535 | 71.5 | Questionnaire | NA | Undefined-reproductible |
| Oliveira dos Santos et al 2021 | LONCAAFS | Boys | Northeast | 636 | NSF | 441 | 69.3 | Questionnaire | NA | Undefined-reproductible |
| Sehn et al 2020 | School Children’s Health | Boys | South | 611 | NSF | 427 | 69.9 | Questionnaire | NA | Undefined-reproductible |
| Sehn et al 2020 | School Children’s Health | Girls | South | 800 | NSF | 588 | 73.5 | Questionnaire | NA | Undefined-reproductible |
| Benedet et al 2013 | NA | Boys | South | 734 | Not reported  ≥8 h/day | 440 | 59.9 | Questionnaire | NA | Undefined |
| Benedet et al 2013 | NA | Girls | South | 832 | Not reported  ≥8 h/day | 527 | 63.3 | Questionnaire | NA | Undefined |
| Guedes et al 2018 | Healthy Paraná Project | Girls | South | 8,776 | Not reported  ≥8 h/day | 2,887 | 32.9 | Questionnaire | NA | Undefined-reproductible |
| Guedes et al 2018 | Healthy Paraná Project | Boys | South | 8,298 | Not reported  ≥8 h/day | 3,037 | 36.6 | Questionnaire | NA | Undefined-reproductible |
| Legnani et al 2015 | NA | Girls | South | 570 | Not reported  ≥10 h/day | 138 | 24.2 | Questionnaire | NA | Undefined |
| Legnani et al 2015 | NA | Boys | South | 484 | Not reported  ≥10 h/day | 82 | 16.9 | Questionnaire | NA | Undefined |
| Noll et al 2013 | NA | Girls | South | 593 | Not reported  8–9 h/day | 320 | 54.0 | Questionnaire | BackPEI | Defined |
| Noll et al 2013 | NA | Boys | South | 744 | Not reported  8–9 h/day | 357 | 48.0 | Questionnaire | BackPEI | Defined |
| Adolescents | | | | | | | | | | |
| Alves et al 2020 | NA | Boys | Northeast | 5,310 | AASM 2016 | 2,235 | 42.1 | Questionnaire | NA | Undefined-reproductible |
| Alves et al 2020 | NA | Girls | Northeast | 6,215 | AASM 2016 | 2,597 | 41.8 | Questionnaire | NA | Undefined-reproductible |
| Caetano 2021 | NA | Boys | Southeast | 133 | AASM 2016 | 31 | 23.3 | Accelerometer | ActiGraph accelerometer (GT3X Model) | Defined |
| Caetano 2021 | NA | Girls | Southeast | 176 | AASM 2016 | 48 | 27.3 | Accelerometer | ActiGraph accelerometer (GT3X Model) | Defined |
| Cardoso et al 2025 | RAPS integration | Boys | Northeast | 1,134 | Not reported  8–10 h/day | 426 | 37.6 | Questionnaire | NA | Undefined-reproductible |
| Cardoso et al 2025 | RAPS integration | Girls | Northeast | 1,791 | Not reported  8–10 h/day | 694 | 38.7 | Questionnaire | NA | Undefined-reproductible |
| Confortin et al 2023 | NA | Boys | Northeast | 1,197.14 | NSF | 544 | 45.5 | Questionnaire | PSQI | Defined |
| Confortin et al 2023 | NA | Girls | Northeast | 1,317.86 | NSF | 578 | 43.9 | Questionnaire | PSQI | Defined |
| De Marco et al 2025 | NA | Girls | South | 477 | Canadian guideline | 223 | 46.9 | Questionnaire | NA | Undefined |
| De Marco et al 2025 | NA | Boys | South | 503 | Canadian guideline | 213 | 42.4 | Questionnaire | NA | Undefined |
| de Oliveira et al 2023 | NA | Girls | Southeast | 248 | NSF | 99 | 39.9 | Questionnaire | NA | Undefined-reproductible |
| de Oliveira et al 2023 | NA | Boys | Southeast | 184 | NSF | 80 | 43.5 | Questionnaire | NA | Undefined-reproductible |
| Domingues et al 2022 | NA | Girls | Southeast | 91 | AASM 2016 | 39 | 42.9 | Accelerometer | Actigraph Gt3x | Defined |
| Domingues et al 2022 | NA | Boys | Southeast | 94 | AASM 2016 | 26 | 27.7 | Accelerometer | Actigraph Gt3x | Defined |
| Maciel. et al 2023 | NA | Boys | Northeast | 639 | Canadian guideline | 247 | 38.7 | Questionnaire | NA | undefined-reproductable |
| Maciel. et al 2023 | NA | Girls | Northeast | 664 | Canadian guideline | 299 | 45.0 | Questionnaire | NA | undefined-reproductable |
| Rocha et al 2024 | ERICA | Girls | Country-wide | 33,255 | Canadian guideline | 14,652 | 44.1 | Questionnaire | NA | Undefined |
| Rocha et al 2024 | ERICA | Boys | Country-wide | 33,536 | Canadian guideline | 15,446 | 46.1 | Questionnaire | NA | Undefined |
| Sehn et al 2022 | School Children’s Health | Boys | South | 626 | NSF | 390 | 62.3 | Questionnaire | NA | Undefined-reproductible |
| Sehn et al 2022 | School Children’s Health | Girls | South | 876 | NSF | 563 | 64.3 | Questionnaire | NA | Undefined-reproductible |
| de Amorim Batista et al 2018 | NA | Boys | Northeast | 221 | Not reported  ≥8 h/day | 67 | 30.3 | Questionnaire | NA | Undefined-reproductible |
| de Amorim Batista et al 2018 | NA | Girls | Northeast | 260 | Not reported  ≥8 h/day | 43 | 16.5 | Questionnaire | NA | Undefined-reproductible |
| dos Santos Araújo et al 2017 | NA | Boys | Northeast | 85 | Not reported  ≥8 h/day | 56 | 65.9 | Questionnaire | NA | Undefined |
| dos Santos Araújo et al 2017 | NA | Girls | Northeast | 135 | Not reported  ≥8 h/day | 92 | 68.1 | Questionnaire | NA | Undefined |
| Felden et al 2016 | NA | Boys | South | 263 | Other reference  ≥8 h/day | 128 | 49.0 | Questionnaire | NA | Undefined |
| Felden et al 2016 | NA | Girls | South | 253 | Other reference  ≥8 h/day | 110 | 43.6 | Questionnaire | NA | Undefined |
| Forket et al 2019 | BRACAH | Girls | South | 540 | NSF | 277 | 51.3 | Questionnaire | NA | Undefined-reproductible |
| Forket et al 2019 | BRACAH | Boys | South | 451 | NSF | 257 | 57.1 | Questionnaire | NA | Undefined-reproductible |
| Meyer et al 2018 | Brazilian Guide for Assessment of Physical Fitness Related to Health and Life Habits | Girls | South | 613 | Other reference  ≥8.33 h/day | 148 | 24.1 | Questionnaire | NA | Undefined |
| Meyer et al 2018 | Brazilian Guide for Assessment of Physical Fitness Related to Health and Life Habits | Boys | South | 519 | Other reference  ≥8.33 h/day | 200 | 38.5 | Questionnaire | NA | Undefined |
| Oliveira et al 2019 | ERICA | Girls | Country-wide | 40,803 | NSF | 33,744 | 82.7 | Questionnaire | NA | Undefined |
| Oliveira et al 2019 | ERICA | Boys | Country-wide | 32,821 | NSF | 26,848 | 81.8 | Questionnaire | NA | Undefined |
| Preschoolers | | | | | | | | | | |
| de Lucena Martins et al 2021 | NA | Girls | Northeast | 138 | Canadian guideline | 92 | 67.0 | Questionnaire | NA | Undefined-reproductible |
| de Lucena Martins et al 2021 | NA | Boys | Northeast | 132 | Canadian guideline | 85 | 65.0 | Questionnaire | NA | Undefined-reproductible |
| Lemos. et al 2024 | NA | Girls | Northeast | 110 | Canadian guideline | 66 | 60.9 | Questionnaire | NA | undefined-reproductable |
| Lemos. et al 2024 | NA | Boys | Northeast | 113 | Canadian guideline | 62 | 54.9 | Questionnaire | NA | undefined-reproductable |
| Martins. et al 2020 | NA | Boys | Northeast | 109 | Canadian guideline | 26 | 24.0 | Questionnaire | NA | undefined-reproductable |
| Martins. et al 2020 | NA | Girls | Northeast | 103 | Canadian guideline | 24 | 24.0 | Questionnaire | NA | undefined-reproductable |
| Mota et al 2025 | Movement's Cool project | Girls | Northeast | 73 | Canadian guideline | 48 | 67.1 | Questionnaire | NA | Undefined-reproductible |
| Mota et al 2025 | Movement's Cool project | Boys | Northeast | 71 | Canadian guideline | 52 | 74.6 | Questionnaire | NA | Undefined-reproductible |

PAAPAS: Parents, Students, Community Health Agents and Teachers for Healthy Eating; LONCAAFS: Longitudinal Study on Sedentary Behavior, Physical Activity, Dietary Habits, and Health of Adolescents; ERICA: Study of Cardiovascular Risks in Adolescents; BRACAH: Brazilian Cardiovascular Adolescent HealtH; BackPEI: Back Pain and Body Evaluation Instrument; PSQI: Pittsburgh Sleep Quality Index

* AASM: American Academy of Sleep Medicine (2016) — Preschoolers (3–5 years): 10–13 h/night; Children (6–12 years): 9–12 h/night; Adolescents (13–18 years): 8–10 h/night; NSF: National Sleep Foundation — Preschoolers (3–5 years): 10–13 h/night; Children (6–13 years): 9–11 h/night; Adolescents (14–17 years): 8–10 h/night; Canadian guidelines: Canadian 24-Hour Movement Guidelines — Early Years (0–4 years): 10–13 h/24 h, including naps (3–4 years); Children and Youth (5–17 years): 9–11 h/night (5–13 years) and 8–10 h/night (14–17 years).

# 6. Supplementary Table S5 - Characteristics of studies reporting continuous sleep duration outcomes for sex

Table 5. Characteristics of studies reporting continuous sleep duration outcomes for sex

| Study label | Study name | Subgroup | Region | Sample size | Mean (SD) | Instrument type | Instrument name | Instrument defined |  |
| --- | --- | --- | --- | --- | --- | --- | --- | --- | --- |
| School-age Children | | | | | | | | | |
| Ferrari. et al 2024 | NA | boys | NA | 446 | 8.7 (1.9) | Questionnaire | NA | undefined-reproductable |  |
| Ferrari. et al 2024 | NA | girls | NA | 429 | 8.7 (1.9) | Questionnaire | NA | undefined-reproductable |  |
| Lemes. et al 2021 | NA | girls | South | 240 | 7.97 (2.41) | Questionnaire | NA | undefined-reproductable |  |
| Lemes. et al 2021 | NA | boys | South | 230 | 7.61 (2.18) | Questionnaire | NA | undefined-reproductable |  |
| Lemes. et al 2021 | NA | girls | Southeast | 555 | 7.4 (1.6) | Questionnaire | NA | undefined |  |
| Lemes. et al 2021 | NA | boys | Southeast | 446 | 7.6 (1.7) | Questionnaire | NA | undefined |  |
| Lemes. et al 2022 | PROESP-BR | girls | South | 240 | 7.97 (2.42) | Questionnaire | MBQ | defined |  |
| Lemes. et al 2022 | PROESP-BR | boys | South | 230 | 7.61 (2.18) | Questionnaire | MBQ | defined |  |
| Roberto et al 2023 | EPOCA | girls | South | 756 | 9.9 (1.06)* | Questionnaire | SSHS | Defined |  |
| Roberto et al 2023 | EPOCA | boys | South | 577 | 9.74 (1)* | Questionnaire | SSHS | Defined |  |
| Sehn et al 2024 | School Children’s Health | boys | South | 1,305 | 9.32 (1.43) | Questionnaire | NA | Undefined-reproductible |  |
| Sehn et al 2024 | School Children’s Health | girls | South | 1,763 | 9.35 (1.43) | Questionnaire | NA | Undefined-reproductible |  |
| Katzmarzyk et al 2015 | ISCOLE | girls | Southeast | 241 | 8.72 (0.85) | Accelerometer | ActiGraph GT3X+ | Defined |  |
| Katzmarzyk et al 2015 | ISCOLE | boys | Southeast | 228 | 8.4 (0.78) | Accelerometer | ActiGraph GT3X+ | Defined |  |
| Adolescents | | | | | | | | | |
| Caetano et al 2024 | NA | girls | Southeast | 176 | 7.53 (0.89)* | Accelerometer | ActiGraph GT3X | Defined |  |
| Caetano et al 2024 | NA | boys | Southeast | 133 | 7.4 (0.74)* | Accelerometer | ActiGraph GT3X | Defined |  |
| da Costa et al 2021 | ELEVA | girls | South | 362 | 6.71 (0.87) | Accelerometer | Actigraph Gt3x+ | Defined |  |
| da Costa et al 2021 | ELEVA | boys | South | 356 | 6.41 (0.78) | Accelerometer | Actigraph Gt3x+ | Defined |  |
| da Costa et al 2021 | ELEVA | girls | South | 375 | 8.05 (1.26) | Questionnaire | NA | Undefined-reproductible |  |
| da Costa et al 2021 | ELEVA | boys | South | 356 | 7.98 (1.34) | Questionnaire | NA | Undefined-reproductible |  |
| de Faria et al 2025 | NA | girls | Southeast | 169 | 6.57 (1.15) | Questionnaire | NA | Undefined-reproductible |  |
| de Faria et al 2025 | NA | boys | Southeast | 180 | 6.7 (1.14) | Questionnaire | NA | Undefined-reproductible |  |
| Domingues et al 2022 | NA | girls | Southeast | 91 | 8.39 (0.93) | Accelerometer | Actigraph Gt3x | Defined |  |
| Domingues et al 2022 | NA | boys | Southeast | 94 | 8.02 (1.04) | Accelerometer | Actigraph Gt3x | Defined |  |
| Santana et al 2022 | 1997/1998 São Luís Birth Cohort | boys | Northeast | 569 | 5.76 (0.94) | Accelerometer | Actigraph wGT3X-BT | Defined |  |
| Santana et al 2022 | 1997/1998 São Luís Birth Cohort | girls | Northeast | 536 | 6.25 (0.94) | Accelerometer | Actigraph wGT3X-BT | Defined |  |
| Collese 2019 | BRACAH | boys | South | 370 | 7.6 (1.96) | Questionnaire | NA | Undefined-reproductible |  |
| Collese 2019 | BRACAH | girls | South | 312 | 7.6 (1.8) | Questionnaire | NA | Undefined-reproductible |  |
| Felden et al 2016 | NA | boys | South | 263 | 8 (1.7) | Questionnaire | NA | Undefined |  |
| Felden et al 2016 | NA | girls | South | 253 | 7.8 (1.5) | Questionnaire | NA | Undefined |  |
| Meyer et al 2018 | Brazilian Guide for Assessment of Physical Fitness Related to Health and Life Habits | girls | South | 613 | 7.6 (1.7) | Questionnaire | NA | Undefined |  |
| Meyer et al 2018 | Brazilian Guide for Assessment of Physical Fitness Related to Health and Life Habits | boys | South | 519 | 7.6 (1.7) | Questionnaire | NA | Undefined |  |
| Schäfer et al 2016 | 1993 Pelotas Birth Cohort | girls | South | 2,080 | 8.8 (1.9) | Questionnaire | NA | Undefined |  |
| Schäfer et al 2016 | 1993 Pelotas Birth Cohort | boys | South | 2,005 | 8.1 (1.8) | Questionnaire | NA | Undefined |  |
| Preschoolers | | | | | | | | | |
| de Lucena Martins et al 2021 | NA | girls | Northeast | 138 | 9.43 (1.11) | Questionnaire | NA | Undefined-reproductible |  |
| de Lucena Martins et al 2021 | NA | boys | Northeast | 132 | 9.45 (1.07) | Questionnaire | NA | Undefined-reproductible |  |
| Goncalves. et al 2022 | NA | girls | Northeast | 170 | 10.22 (1.5) | Questionnaire | NA | undefined |  |
| Goncalves. et al 2022 | NA | boys | Northeast | 154 | 10.25 (1.37) | Questionnaire | NA | undefined |  |
| Lemos. et al 2024 | NA | girls | Northeast | 110 | 10.1 (1.3) | Questionnaire | NA | undefined-reproductable |  |
| Lemos. et al 2024 | NA | boys | Northeast | 113 | 10.1 (1.4) | Questionnaire | NA | undefined-reproductable |  |
| Mota et al 2025 | Movement's Cool project | girls | Northeast | 73 | 8.93 (1.06) | Questionnaire | NA | Undefined-reproductible |  |
| Mota et al 2025 | Movement's Cool project | boys | Northeast | 71 | 9.2 (1.44) | Questionnaire | NA | Undefined-reproductible |  |

PROESP-BR: Projeto Esporte Brasil; ISCOLE: International Study of Childhood Obesity, Lifestyle and the Environment; ELEVA: Longitudinal Study of the Lifestyle of Adolescents; BRACAH: Brazilian Cardiovascular Adolescent Health; MBQ: Movement Behavior Questionnaire; SSHS: School Sleep Habits Survey.

# 7. Supplementary Figure S1 – Forest plot Age X Instrument (Categorical)

# 8. Supplementary Figure S2 – Forest plot Age x Sex (Categorical)

# 9. Supplementary Figure S3 – Forest plot Age x Guideline (Categorical)

# 10. Supplementary Figure S4 – Forest plot Age x Sex (Continuous)

# 11. Supplementary Figure S5 – Forest plot Age x Instrument (Continuous)

# 12. **Supplementary Figure S6 – Funnel plot (Categorical outcome)**

# 13. Supplementary Figure S7 – Funnel plot (Continuous outcome)
